# The dynamic and diverse nature of parenchyma cells in the *Arabidopsis* root during secondary growth

**DOI:** 10.1038/s41477-025-01938-6

**Published:** 2025-03-26

**Authors:** Munan Lyu, Hiroyuki Iida, Thomas Eekhout, Meeri Mäkelä, Sampo Muranen, Lingling Ye, Anne Vatén, Brecht Wybouw, Xin Wang, Bert De Rybel, Ari Pekka Mähönen

**Affiliations:** 1 Organismal and Evolutionary Biology Research Programme, Faculty of Biological and Environmental Sciences and Viikki Plant Science Centre, University of Helsinki, Helsinki, Finland; 2 Department of Plant Biotechnology and Bioinformatics, Ghent University, Ghent, Belgium; 3 VIB Centre for Plant Systems Biology, Ghent, Belgium; 4 VIB Single Cell Core, VIB, Ghent, Belgium; 5 VIB Single Cell Core, VIB, Leuven, Belgium

**Keywords:** Plant morphogenesis, Cell fate, Wounding, Jasmonic acid

## Abstract

During secondary growth, the vascular cambium produces conductive xylem and phloem cells, while the phellogen (cork cambium) deposits phellem (cork) as the outermost protective barrier. Although most of the secondary tissues are made up of parenchyma cells, which are also produced by both cambia, their diversity and function are poorly understood. Here we combined single-cell RNA sequencing analysis with lineage tracing to recreate developmental trajectories of the cell types in the *Arabidopsis* root undergoing secondary growth. By analysing 93 reporter lines, we were able to identify 20 different cell types or cell states, many of which have not been described before. We additionally observed distinct transcriptome signatures of parenchyma cells depending on their maturation state and proximity to the conductive cell types. Our data show that both xylem and phloem parenchyma tissues are required for normal formation of conductive tissue cell types. Furthermore, we show that mature phloem parenchyma gradually obtains periderm identity, and this transformation can be accelerated by jasmonate treatment or wounding. Our study thus reveals the diversity of parenchyma cells and their capacity to undergo considerable identity changes during secondary growth.

## Abstract (web summary)

A combination of lineage tracing and single-cell RNA sequencing reveals how xylem and phloem parenchyma cells in the secondary tissue of *Arabidopsis* root mature gradually. Upon root barrier injury, this maturation involves a change in cell identity from phloem parenchyma to barrier cell types.

## Main

Besides primary growth in the apical meristems, many seed plants exhibit secondary (that is, radial) growth in their mature stems and roots. This process is orchestrated by lateral meristems, the vascular cambium and the phellogen (cork cambium). The vascular cambium produces conductive cells, secondary xylem inward and secondary phloem outward, and the phellogen provides a barrier tissue, the phellem (cork) outward. Besides these conductive or barrier cell types, the vascular and cork cambia also generate xylem and phloem parenchyma cells, and the phelloderm, respectively. These parenchymatic cells occupy the largest part of the secondary tissues^1–3^. Parenchyma consists of thin-walled living cells, and in many plant species, it is clear that these parenchyma cells have different functions^1^. Although mesophyll cells, for example, are essential for photosynthesis, the function of most parenchyma cells remains unknown. In the secondary tissues, this is probably because parenchyma cells have received less attention than the conductive cells. Although a few reports suggest heterogeneity of xylem parenchyma cells^4^, it has not been proved experimentally, and it is unclear whether parenchyma cells with similar morphological features within the same tissue have a heterogenous or homogeneous identity. Here we optimized single-cell RNA sequencing (scRNA-seq)^5–7^ on *Arabidopsis* mature roots to explore the diversity of cell types in roots undergoing secondary growth. We confirmed that our dataset contains all known conductive and parenchymatic cell types in *Arabidopsis* secondary tissues and demonstrate that xylem and phloem parenchyma are composed of diverse cell types and cell states. Through extensive reporter analysis combined with mutant analysis, we also found that the xylem and phloem parenchyma cells function in supporting conductive tissue formation. Furthermore, lineage tracing analysis suggests that mature phloem parenchyma cells can change their cell identity to replenish the barrier upon injury. Taken together, our study demonstrates the diverse and dynamic nature of parenchyma cells in *Arabidopsis* secondary tissues.

## Results

### Single-cell RNA-seq reveals diverse cell types in mature roots

**Fig. 1:**
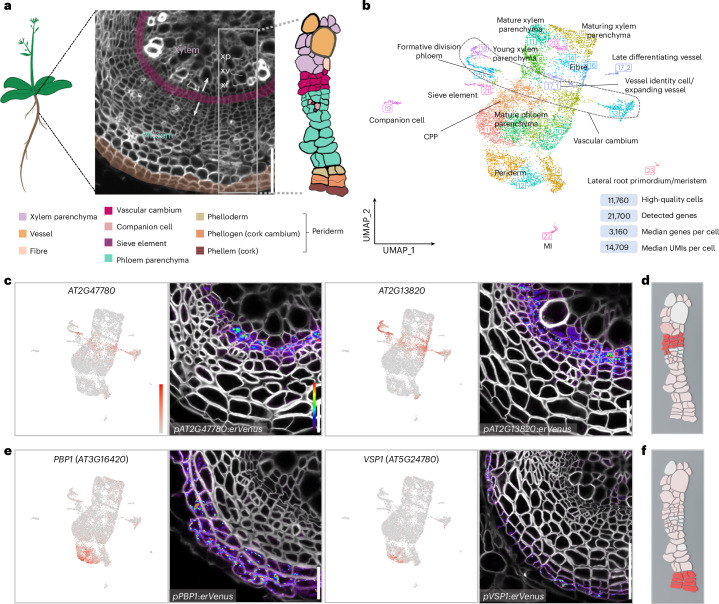
Single-cell transcriptome profiling of the *Arabidopsis* mature root. **a**, The tissue used for scRNA-seq. A cross-section of 30-day-old *Arabidopsis* mature root is shown. The arrows point in the direction of vascular cambium proliferation. xp, xylem parenchyma; xv, xylem vessel; xf, xylem fibre. **b**, Visualization of 23 cell clusters using UMAP, with identity annotations validated by reporter analysis. Each dot represents an individual cell, with colours representing different clusters. The dashed outline highlights the vascular cambium cells. The dataset quality information is displayed in the bottom right corner. UMIs, unique molecular identifiers. **c**, UMAP plots of *AT2G47780* and *AT2G13820* specifically detected in vascular cambium clusters and confocal cross-sections of their promoter–reporter lines. **d**, Expression of *AT2G13820* in the Cella model. **e**, UMAP plots of *PBP1* and *VSP1* highly detected in periderm clusters and confocal cross-sections of their promoter–reporter lines. **f**, Expression of *VSP1* in the Cella model. In the cross-sections in **a**, **c** and **e**, cell walls were stained with SR2200. In **c** and **e**, for each gene, the UMAP plot is shown on the left, and the cross-section of a 16-day-old root is shown on the right. The relative expression levels of genes in UMAP and Venus–YFP signals in the cross-sections are shown according to the colour scale in **c**. Detailed information on the reporters is available in Supplementary Table 4. Scale bars, 100 µm (**a**), 20 µm (**c**,**e**).

**Extended Data Fig. 1:**
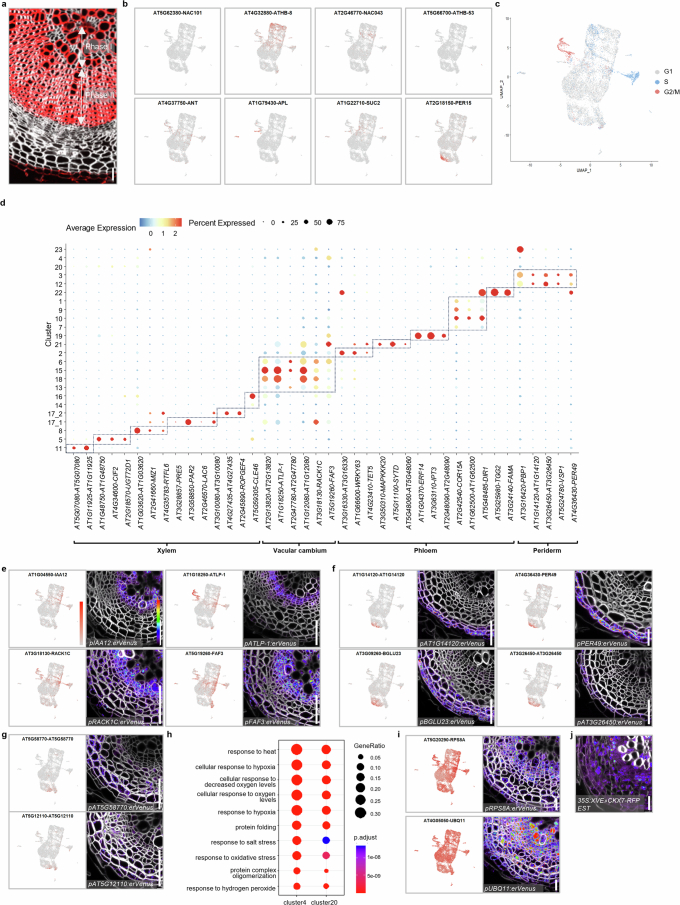
An overall analysis of the single cell RNA-seq data. **a**, Confocal cross-section of six-week-old *Arabidopsis* mature root. Cell wall stained with SR2200 were shown in grey, lignin stained with Basic Fuchsin was shown in red. **b**, UMAP plots of known tissue-specifically expressed genes (*NAC101, ATHB8, NACO43, ATHB53, ANT, APL, SUC2*, and *PER15*). **c**, Predicted classification of each cell in either G2/M, S or G1 phase in UMAP. Cell cycle list from Zhang et al., 2019 was utilized. **d**, Dotplot of expression of representative marker genes used for cluster annotation and data validation. Size or colour of dots represents the percentage of cells expressing a given gene or the scaled average expression level, respectively. Box with dotted line represents tissue types. **e**, UMAP plots of genes (*IAA12, ATLP-1, RACK1C* and *FAF3*) highly detected in vascular cambium clusters and confocal cross-sections of their transcriptional reporter lines. **f**, UMAP plots of genes (*AT1G14120, PER49, BGLU23* and *AT3G26450*) highly detected in periderm clusters and confocal cross-sections of their transcriptional reporter lines. **g**, UMAP plots of genes (*AT5G58770* and *AT5G12110*) highly detected in cluster 4 and 20, and confocal cross-sections of their transcriptional reporter lines. **h**, Gene Ontology (GO) enrichment analysis and comparison of differentially expressed gene (DEG)s of cluster 2,6,13,15 and 18. Top 12 enriched biological processes are shown. Dot size represents percentage of DEGs in a given cluster involved in the biological process; dot color indicates P.adjust value of each biological process in a given cluster. The full list of GO terms of all the clusters is available in Supplementary Table 3. The GO enrichment test was performed based on two-sided hypergeometric distribution. The p values were adjusted for multiple comparisons. **i**, UMAP plots of genes (*RPS8A* and *UBQ11*) broadly and highly detected in all clusters and confocal cross-sections of their transcriptional reporter lines. **j**, Confocal microscopy of cross-section of 13-day-old *35S:XVE* » *CKX7-RFP* root grown with 5 μM estrodiol (EST) for 1day. Cell wall stained with calcofluor was shown in grey. The experiment was independently repeated three times. In **e**-**g** and **i**, for each gene, the UMAP plot was shown in left and cross section was shown in right. **e**-**g** and **i** show confocal cross-sections of 16-day-old roots. The detailed information of reporters is available in Supplementary Table 4. Cell walls stained with SR2200 were shown in grey. Relative expression levels of genes in UMAP and Venus-YFP signals in cross sections were shown according to the color map in **e**. Scale bars, 20 µm (**e**-**f**, **h**).

To explore cell type diversity during root secondary growth, we produced an scRNA-seq atlas. Secondary growth in the hypocotyl and root of *Arabidopsis* can be divided into two distinct phases. In phase I, vascular cambium produces xylem parenchyma and vessels, and in phase II, vessels and fibres^8^ (Extended Data Fig. 1a). For this study, we used 30-day-old *Arabidopsis* roots that were transitioning from phase I to II and thus producing all three xylem cell types, parenchyma, fibres and vessels (Fig. 1a and Extended Data Fig. 1a). Sections of the first 2 cm below the root–hypocotyl junction were collected for protoplast isolation and subsequent transcriptome profiling (Fig. 1a). After quality control^6^, 11,760 high-quality cells (with a median of 3,160 genes and a median of 14,709 reads per cell) were retained and visualized using uniform manifold approximation and projection (UMAP)^9^ (Fig. 1b and Supplementary Tables 1–3). To examine whether the major cell types known to be present in this tissue were captured in our dataset, we initially analysed the expression of known tissue-specific genes^10–17^. On the basis of these markers, all the known cell types were predicted to be present in the dataset, while several clusters remained unidentified (Extended Data Fig. 1b,c). The relative positions of each known cell type in the dataset UMAP reflect the real organization of the secondary tissue, indicating that we probably captured most of the developmental states and transitions between them (Fig. 1a,b). To validate these predictions and annotate all the remaining clusters, we examined the expression of 93 reporters: 16 published before^18–25^ and 77 generated for this study (Supplementary Table 4 and Extended Data Fig. 1d). Many of these reporters indicate cell states for which no marker had been previously identified and can serve as tissue-specific markers in secondary tissues of *Arabidopsis* roots. Only genes highly expressed in two clusters showed no correlation with specific cell stage or state on the basis of reporter analysis (Extended Data Fig. 1g,h). A detailed description of the 93 reporter lines used to annotate the clusters is provided below, as well as in Supplementary Table 4 and Supplementary Notes. Strong and ubiquitous promoters useful for overexpression studies in the secondary tissue are also presented in Supplementary Notes and in Extended Data Fig. 1i,j.

**Extended Data Fig. 2:**
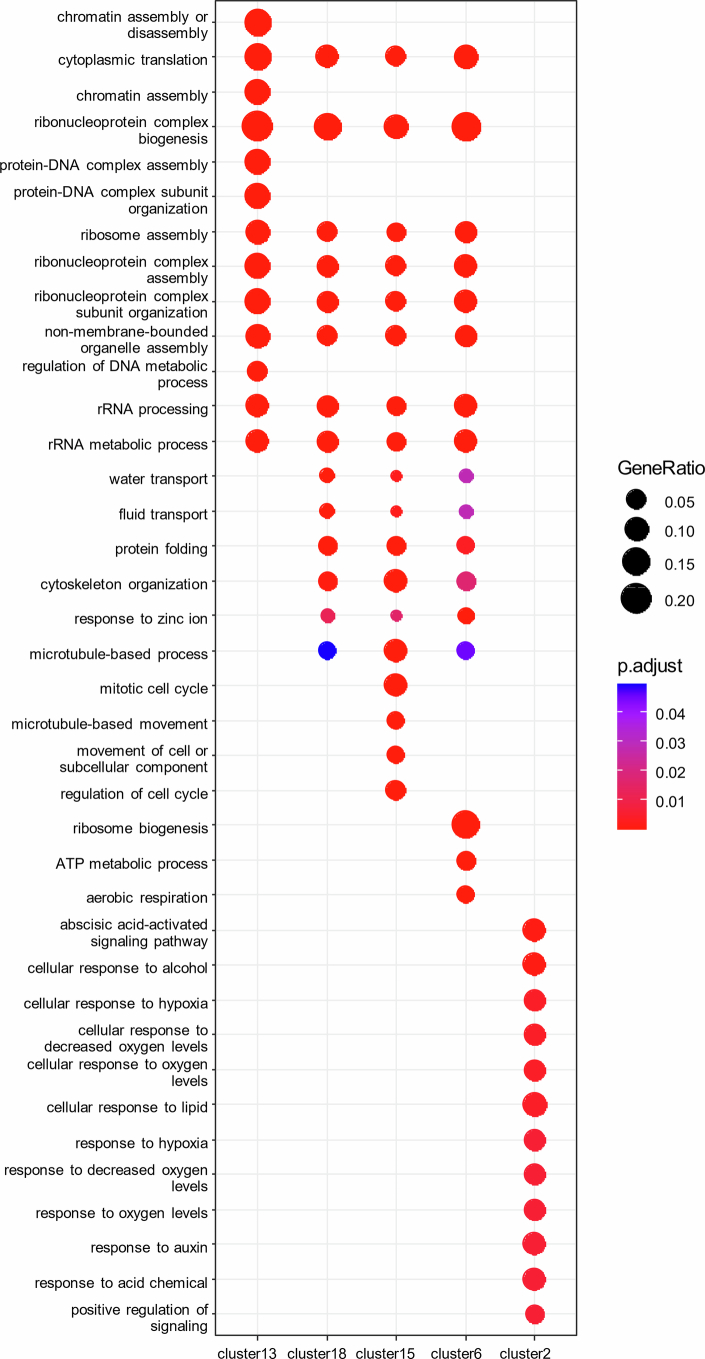
GO term comparison among vascular cambium clusters. GO enrichment analysis and comparison among DEGs of cluster 2, 6, 13, 15 and 18. Top 12 enriched biological processes are shown. Dot size represents percentage of DEGs in a given cluster involved in the biological process; dot color indicates P.adjust value of each biological process in a given cluster. The full list of GO terms of all the clusters is available in Supplementary Table 3. The GO enrichment test was performed based on two-sided hypergeometric distribution. The p values were adjusted for multiple comparisons.

Vascular cambium and phellogen (cork cambium) are secondary meristems that orchestrate radial growth^1^. Since some key vascular cambium regulators are expressed in the periderm (that is, phellem, phellogen and phelloderm) as well, these two cambia share core developmental regulators^2^. However, the different functions of these two cambia also indicate that each has its specific regulators. Our scRNA-seq data captured numerous cells of both cambia and successfully identified genes uniquely expressed in either cambium, as well as those expressed in both (Fig. 1c–f, Extended Data Figs. 1e,f and 2 and Supplementary Notes). We generated and validated a transcriptome atlas of the *Arabidopsis* root undergoing secondary growth, allowing us to reveal shared and unique regulatory mechanisms driving vascular cambium and/or phellogen activity. This atlas is available in an online tool (https://single-cell.be/plant/root-secondary-tissue) together with a model based on Cella^26^, a visualization tool that maps single-cell-resolution gene expression data onto the plant model. This platform is user-friendly, making it more accessible for non-specialists to explore gene expression data (Fig. 1d,f).

### Three maturation states in xylem parenchyma

Secondary growth occurs almost exclusively in the radial dimension. It provides easily distinguishable radial cell files, from which the developmental progression of cells can be deduced by observing specific morphological features or the relative distance from the vascular cambium. This feature has been used, for example, when developmental and transcriptional progression of xylem cells with secondary cell walls has been profiled using cryosectioning^27^. We used the same logic here to identify the developmental progression of different xylem and phloem cell types as they exit the vascular cambium. Thus, when genes enriched in distinct clusters showed expression in the same cell type but at different distances from the vascular cambium, we deduced that these two clusters represent different maturation states of that cell type.

**Extended Data Fig. 3:**
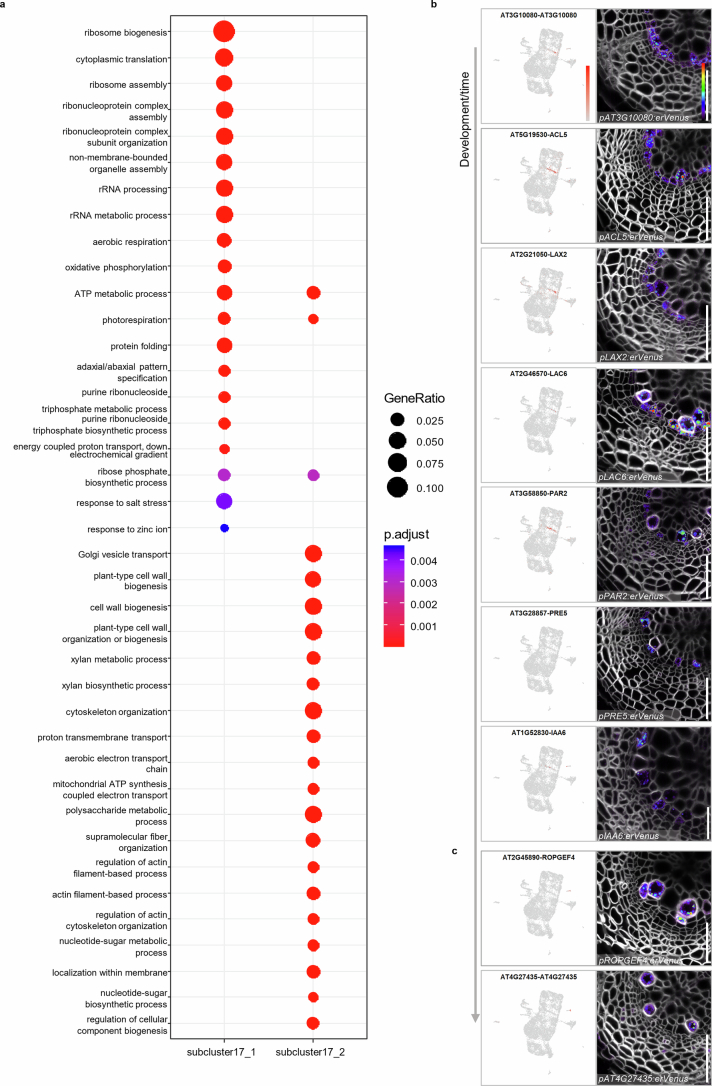
Distinct states during vessel development. **a**, GO enrichment analysis and comparison of DEGs of subcluster 17_1 and 17_2. Dotplot shows top 20 enriched biological processes. Dot size represents percentage of DEGs in a given cluster involved in the biological process, dot color indicates P.adjust value of each biological process in a given cluster. The full list of GO terms of both subclusters is available in Supplementary Table 3. The GO enrichment test was performed based on two-sided hypergeometric distribution. The p values were adjusted for multiple comparisons. **b**, UMAP plots of genes (*AT3G10080*, *ACL5, LAX2, LAC6, PAR2, PRE5* and *IAA6*) specifically detected in vessel identity cell / expanding vessel cluster and confocal cross-sections of their transcriptional reporter lines. **c**, UMAP plots of genes (*ROPGEF4* and *AT4G27435*) specifically detected in late differentiating vessel cluster and confocal cross-section of their transcriptional reporter lines. In **b**, **c**, for each gene, the UMAP plot was shown in left and cross section of 16-day-old root was shown in right. Cell walls stained with SR2200 were shown in grey. Relative expression levels of genes in UMAP and Venus signals in cross sections were shown according to the colour map in **b**. The detailed information of reporters is available in Supplementary Table 4. Scale bars, 20 µm (**b**, **c**).

**Extended Data Fig. 4:**
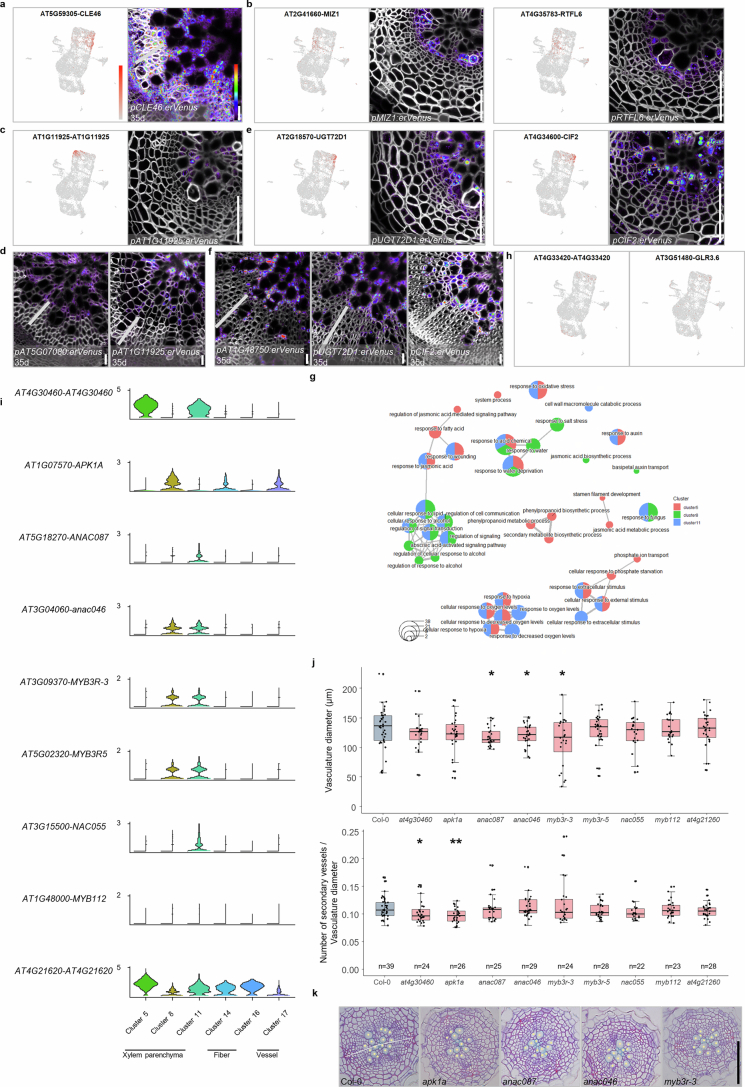
Analysis of xylem fiber and parenchyma clusters. **a**, UMAP plot of *CLE46* highly detected in differentiating fiber clusters and confocal cross-section of its transcriptional reporter line. **b**, UMAP plots of genes (*MIZ1* and *RTFL6)* highly detected in young xylem parenchyma cells and confocal cross-sections of their transcriptional reporter lines. **c**, UMAP plot of *AT1G11925* highly detected in mature xylem parenchyma cells and confocal cross-sections of its transcriptional reporter line. **d**, Confocal cross-sections of transcriptional reporter lines (*AT5G07080* and *AT1G11925*). **e**, UMAP plots of genes (*UGT72D1* and *CIF2*) highly detected in maturing xylem parenchyma cells and confocal cross-sections of their transcriptional reporter lines. **f**, Confocal cross-sections of transcriptional reporter lines (*AT1G48750, UGT72D1* and *CIF2*). In **a**-**c** and **e**, for each gene, the UMAP plot was shown in left and cross section was shown in right. Relative expression levels of genes in UMAP and Venus signals in cross sections were shown according to the colour map in **a**. The detailed information of reporters is available in Supplementary Table 4. Cross sections of **b, c, e** were from 16-day-old roots. Cross sections of **a**, **d**, **f** were from 35-day-old roots. In **a**, **d**, **f**, region of xylem fiber was marked with grey bar. In cross sections of **a**-**f**, cell walls stained with SR2200 were shown in grey. In **d**, **f**, more than five T1 individuals were imaged. **g**, GO enrichment analysis and comparison among DEGs of cluster 5, 8 and 11 shown with emapplot. The top 15 enrich biological processes were shown. Dot size represents the number of DEGs in a given cluster involved in the biological process. The edge represents the similarity between terms; shorter and thicker edge indicates higher similarity. The full list of GO terms of all clusters is available in Supplementary Table 3. **h**, UMAP plots of *PRX47*/*AT4G33420* and *GLR3.6*. **i**, Violin plots of genes highly expressed in xylem parenchyma clusters. The height and width of the plots represent the expression level and the proportion of cells showing expression, respectively. **j**, Quantification of vasculature diameter and ratio between secondary vessel numbers and vasculature diameter in 14-day-old seedlings in which each gene shown in **i** was mutated. The boxes in the box-and-whisker plots represent median values and interquartile range, the whiskers indicate the total range. The black dots indicate measurements from individual roots. Shapiro-Wilk normality test followed by a two-sided Wilcoxon test was utilized for statistic test between Col-0 and mutants. *p < 0.05, **p < 0.01. *p*_*anac087_diameter*_ = 0.01061639, *p*_*anac046_diameter*_ = 0.03029816, *p*_*myb3r-3_diameter*_ = 0.03014993, *p*_*at4g30460_vessel/diameter*_ = 0.03614403, *p*_*apk1a_ vessel/diameter*_ = 0.003126792. The experiment was repeated twice; n indicated number of individuals analysed. **k**, Bright-field cross sections of 14-day-old wildtype (Col-0), *apk1a*, *anac087, anac046*, and *myb3r*-3 single mutant roots. Yellow dots indicate secondary vessels. White line indicates the vasculature diameter based on primary xylem and two primary phloem poles. Scale bars, 20 µm (**a**-**f**), 50 µm (**k**). Source data

We next analysed different cell types within the xylem domain by combining bioinformatic and reporter analyses. We identified xylem fibres and xylem vessels at various stages of development in the dataset. Subcluster 17_1 represents cells that have recently obtained vessel identity and initiated cell expansion (Supplementary Notes and Extended Data Fig. 3a,b) and are thus expressed in the xylem side of the cambium. From subcluster 17_1, cells progress to subcluster 17_2 to finalize cell expansion and undergo terminal differentiation, including secondary cell wall formation (Supplementary Notes and Extended Data Fig. 3a,c). Clusters 14 and 16 represent fibre cells, marked with peak expression of the *CLAVATA3/ESR (CLE)-RELATED PROTEIN 46* (*CLE46*) reporter during phase II when fibres are being produced (Supplementary Notes and Extended Data Fig. 4a).

**Fig. 2:**
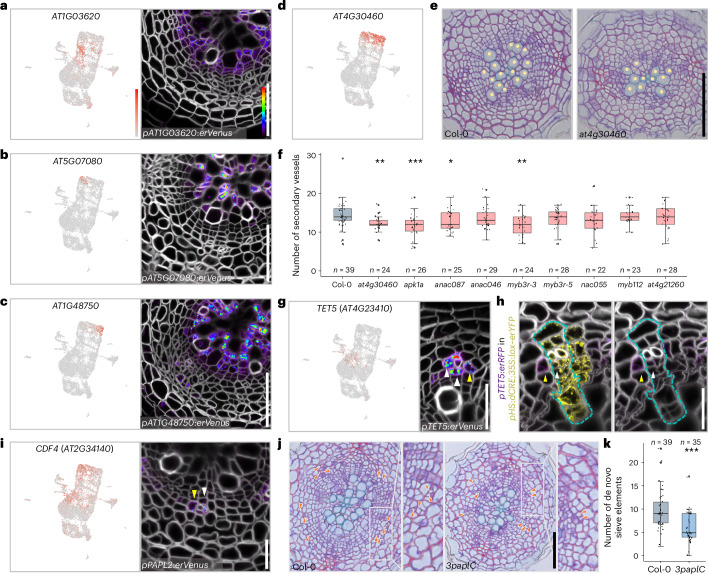
Diversity of parenchyma cells and their functions in conductive tissue development. **a**–**c**, Expression of *AT1G03620* (**a**), *AT5G07080* (**b**) and *AT1G48750* (**c**) in young, mature and maturing xylem parenchyma clusters, respectively; and confocal cross-sections of their promoter–reporter lines. **d**, Expression of *AT4G30460* in xylem parenchyma clusters. **e**, Bright-field cross-sections of 14-day-old wild-type and *at4g30460* single-mutant roots. The yellow dots indicate secondary vessels. **f**, Quantification of secondary vessel number in 14-day-old wild-type and mutant seedlings. **g**, Expression of *TET5* in the CPP cluster and confocal cross-section of its promoter–reporter line. **h**, Confocal cross-section of 16-day-old *pTET5*:*erRFP* in *pHS*:*dCRE*;*35S*:*lox–erYFP* root 6 days after clone induction. Left: merged image. Right: image with RFP and cell wall channels. A sector from a single clone is enclosed in dashed lines. The experiment was independently repeated three times. **i**, Expression of *CDF4*/*PAPL2* and confocal cross-sections of its promoter–reporter line. In **g**–**i**, the white and yellow arrowheads indicate CPP cells originating from formative division and recruited from neighbouring cell lineages, respectively. **j**, Bright-field cross-sections of 16-day-old wild-type and *3paplC* mutant roots. The orange arrowheads point at de novo sieve elements, which appear lightly stained adjacent to the intensively purple-stained companion cells. **k**, Quantification of de novo sieve element number in 16-day-old wild-type and *3paplC* mutant seedlings. In **a**–**c**, **g** and **i**, for each gene, the UMAP plot is shown on the left, and the cross-section of a 16-day-old root is shown on the right. Detailed information on the reporters is available in Supplementary Table 4. In the cross-sections in **a**–**c** and **g**–**i**, cell walls were stained with SR2200. In **f** and **k**, the boxes in the box-and-whisker plots represent the median values and interquartile range, and the whiskers indicate the total range. The black dots indicate measurements from individual roots. A Shapiro–Wilk normality test followed by a two-sided Wilcoxon test was used to test for differences between Col-0 and the mutants. *P < 0.05; **P < 0.01; ***P < 0.001. *P*_*at4g30460*_vessel_ = 0.001833701; *P*_*apk1a*_vessel_ = 0.0008894759; *P*_*anac087*_vessel_ = 0.04769388; *P*_*myb3r-3*_vessel_ = 0.00422161; *P*_*3paplC*_sieve_element_ = 0.0002414. The experiment was repeated twice (**f**) or four times (**k**). *n* indicates the number of examined roots. The relative expression levels of genes in UMAP and Venus–YFP signals in the cross-sections are shown according to the colour scale in **a**. Scale bars, 50 µm (**e**,**j**), 20 µm (**a**–**c**,**g**–**i**). Source data

Given that xylem parenchyma is less comprehensively understood than the morphologically distinct xylem cell types, we conducted a deeper analysis on xylem parenchyma. We found that xylem parenchyma cells were classified in three clusters (cluster 8, 11 and 5) containing different transcriptional profiles. The transcriptional reporter lines of genes predominantly expressed in cluster 8 (*ROTUNDIFOLIA LIKE 6* (*RTFL6*), *MIZU-KUSSEI 1* (*MIZ1*) and *AT1G03620*) showed high fluorescence signals in young xylem parenchyma cells adjacent to the meristematic zone and near the expanding vessels (Fig. 2a and Extended Data Fig. 4b). Reporter expression driven by the promoters of cluster-11-enriched genes (*AT5G07080* and *AT1G11925*) were preferentially detected in the most mature xylem parenchyma cells near the primary xylem axis in both 16-day-old (phase I) and 35-day-old (phase II) roots (Fig. 2b and Extended Data Fig. 4c,d). The promoter activities of cluster-5-enriched genes (*AT1G48750*, *UDP-GLUCOSYL* *TRANSFERASE* *72D1* (*UGT72D1*) and *CASPARIAN STRIP INTEGRITY FACTOR 2* (*CIF2*)) were detected in the parenchyma between the mature and young xylem parenchyma cells, which we termed ‘maturing xylem parenchyma’. In phase II roots, these reporters were also present in cells adjacent to vessels (Fig. 2c and Extended Data Fig. 4e,f). These results thus suggest that the xylem parenchyma is not a homogenous tissue but is composed of cells in different maturation states. To further characterize the different states, we performed Gene Ontology (GO) comparison of xylem parenchyma clusters. Cluster 8 showed specific responses to salt stress and auxin transport, and cluster 11 to hypoxia. Both clusters were also enriched by genes related to the response to fungi (Extended Data Fig. 4g). Differentially expressed genes in cluster 5 were predicted to be involved in phenylpropanoid biosynthesis and metabolic pathways that contribute to processes such as lignin biosynthesis and defence responses^28^ (Extended Data Fig. 4g). Vessel lignification occurs through both cell-autonomous process in the differentiating vessels and non-cell-autonomous process by the adjacent xylem parenchyma cells, known as ‘good neighbour’ cells^29,30^. It appears that maturing xylem parenchyma corresponds to the good neighbour cells, since a specific reporter for these cells, *PEROXIDASES47* (*AT4G33420*)^30^, shows peak expression in cluster 5 (Extended Data Fig. 4h). Additionally, wound-induced electrical signalling propagates in part through ‘xylem contact’ cells in leaf veins^31^. The mediator of the electric signal, GLR3.6 (a glutamate receptor-like protein^31^), shows peak expression in cluster 5 in our dataset (Extended Data Fig. 4h), implying a role of maturing xylem parenchyma in stress responses. Our study thus suggests that the maturing xylem parenchyma cells are associated with both wound signalling and vessel lignification.

To further investigate the function of xylem parenchyma cells, we examined a series of T-DNA insertion mutants whose corresponding genes are expressed preferentially in any of the states of xylem parenchyma maturation in the UMAP (Fig. 2d and Extended Data Fig. 4i). Even though the expression of these genes was low in the vessel cluster, nearly half of the examined single mutants (four out of nine mutants) showed a significant reduction in secondary vessel formation (Fig. 2e,f and Extended Data Fig. 4j,k). Indeed, secondary vessel formation was significantly decreased in the *at4g30460* and *arabidopsis protein kinase 1* (*apk1a*) loss-of-function mutants (Fig. 2e,f and Extended Data Fig. 4j,k), even when the reduced secondary growth in these mutants was taken into account. Taken together, our results indicate the existence of a diverse range of xylem parenchyma cells, and that one of their potential functions is to promote vessel formation during secondary growth. However, further studies are needed to explore how direct the link between xylem parenchyma and vessel production is.

### Parenchyma cells that control conductive phloem formation

**Extended Data Fig. 5:**
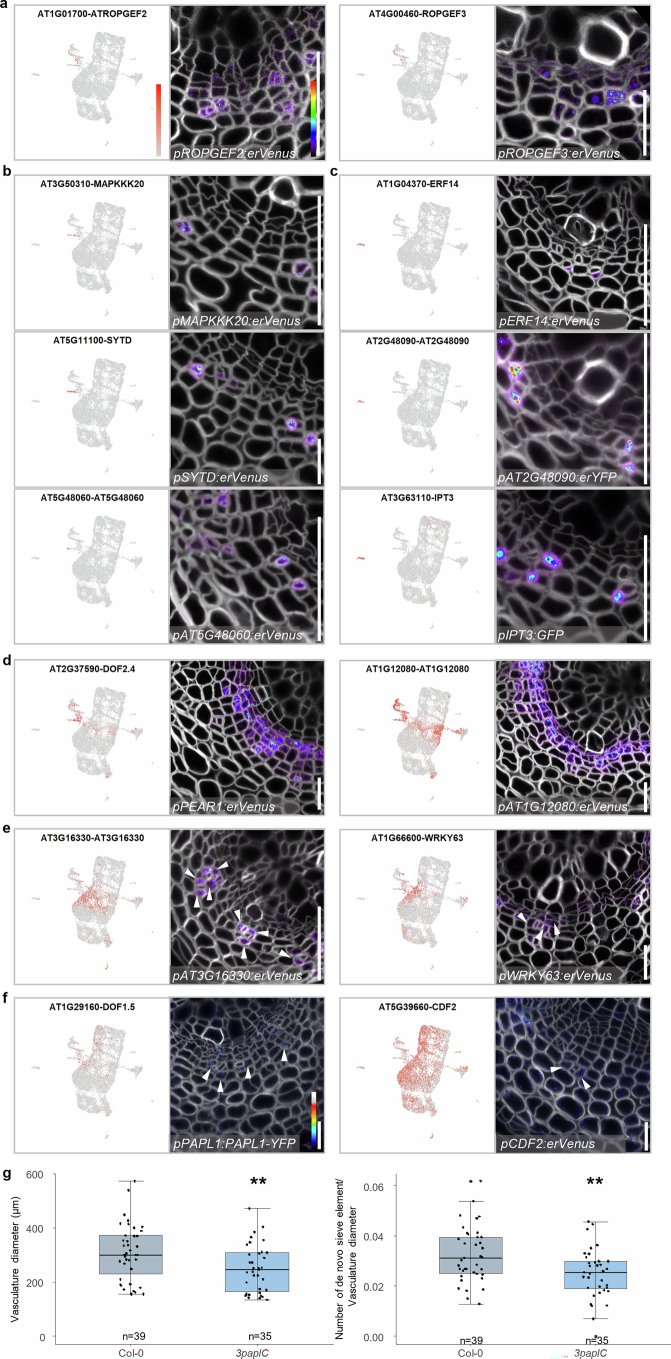
Analysis of phloem clusters. **a**, UMAP plots of genes *(ROPGEF2* and *ROPGEF3*) highly detected in formative division phloem clusters and confocal cross-sections of their transcriptional reporter lines. **b**, UMAP plots of genes (MAPKKK20, *SYTD* and *AT5G48060*) specifically detected in differentiating sieve element cluster and confocal cross-sections of their transcriptional reporter lines. **c**, UMAP plots of genes (*ERF14*, *AT2G48090* and *IPT3*) specifically detected in companion cell cluster and confocal cross-sections of their transcriptional reporter lines. **d**, UMAP plots of genes (*DOF2.4*/*PEAR1* and *AT1G12080*) highly detected in phloem side cambium clusters and confocal cross-section of their transcriptional reporter lines. **e**, UMAP plots of genes (*AT3G16330* and *WRKY63*) highly detected in CPP cluster and confocal cross-sections of their transcriptional reporter lines. **f**, UMAP plots of *PAPL* genes (*DOF1.5*/ *PAPL1* and *CDF2*) and confocal cross-sections of their reporter lines. Relative expression levels of Venus signals in cross sections were shown according to the colour map. In **e**, **f**, white arrowheads indicate CPP cells. In **a-f**, for each gene, the UMAP plot was shown in left and cross section was shown in right. The detailed information of reporters is available in Supplementary Table 4. **a-f** show confocal cross-sections of 16-day-old roots. Cell walls stained with SR2200 were shown in grey. Relative expression levels of genes in UMAP (**a**-**f**) and *Venus* signals in cross sections (**a**-**e)** were shown according to the colour map in **a**. **g**, Quantification of vasculature diameter and ratio between number of *de novo* sieve elements and vasculature diameter of 16-day-old wildtype and *3paplC* roots. The boxes in the box-and-whisker plots represent median values and interquartile range, the whiskers indicate the total range. The black dots indicate measurements from individual roots. Shapiro-Wilk normality test followed by a two-sided Wilcoxon test was utilized for statistic test between Col-0 and *3paplC*. **p < 0.01. *p*_*3paplC_diameter*_ = 0.04647, *p*_*3paplC_sieve_element/diameter*_ = 0.004744. The experiment was repeated 4 times, thirty-nine wildtype seedlings and thirty-five *3paplC* seedlings were examined in total. Scale bars, 20 µm (**a-f**). Source data

We next investigated the cell types within the phloem domain by integrating bioinformatic and reporter analysis with lineage tracing. Like the primary phloem differentiation process in the root tip^18,19^, the formative division in phloem identity cells (a subgroup of cluster 15) gives rise to sieve elements (cluster 21) and companion cells (cluster 19) (Supplementary Notes and Extended Data Fig. 5a–c).

Since most secondary phloem tissue consists of parenchyma cells (Fig. 1a) but besides this we know very little about this cell type, we carried out detailed analysis of the phloem parenchyma cell clusters. During the validation process, we noticed that promoter activities of the phloem-side cambium genes, *DNA BINDING WITH ONE FINGER 2.4* (*DOF2*.4) / *PHLOEM EARLY DOF* (*PEAR1*)^11,18^ and *AT1G12080*, were also highly detected in parenchyma cells adjacent to the sieve elements and companion cells (Extended Data Fig. 5d). This expression implies the unique identity of phloem parenchyma associated with conductive phloem cells. This type of expression pattern seems to be associated with cluster 2 since the reporters of cluster-2-enriched genes (*TETRASPANIN5* (*TET5*), *AT3G16330* and *WRKY DNA-BINDING PROTEIN 63* (*WRKY63*) showed preferential expression in those parenchyma cells (Fig. 2g and Extended Data Fig. 5e). We named these cells in cluster 2 conductive phloem-associated parenchyma (CPP) cells. Next, we studied the ontogeny of these CPP cells and devised two possible mechanisms. First, CPP identity could be established via lineage-based mechanisms by which these cells are coordinately formed with the conductive phloem within the same lineage. Alternatively, these cells could be recruited by cell–cell-communication-based mechanisms from neighbouring conductive cells regardless of their lineage. To examine these two possibilities, we generated a *pTET5*:*erRFP* reporter line in the background of the cell-lineage-tracing line in which the clones were marked with the expression of the *YFP* reporter gene upon heat shock (modified from Smetana et al.^11^). Sector analysis was performed 6 days after *YFP* clones were induced in 10-day-old roots. The *pTET5*:*erRFP* signal was detected in CPP cells of both the conductive and non-conductive phloem lineages (Fig. 2h). CPP identity is thus obtained because of recruitment by neighbouring conductive phloem cells in a cell-non-autonomous manner. However, we cannot exclude the possibility that a proportion of CPP cells are formed cell-autonomously as a result of formative cell divisions within the conductive phloem lineage. The location of the CPP cells also reminded us of genes expressed in the cells surrounding the conductive phloem in primary roots such as *PINEAPPLE* (*PAPL*) transcription factors^20^. In our UMAP, three *PAPL* genes showed high expression in cluster 2, and, consistently, fluorescence signals in their reporter lines were detected in CPP cells (Fig. 2i and Extended Data Fig. 5f). While there was a slight but significant reduction in secondary growth in the *papl* triple mutant (Fig. 2j and Extended Data Fig. 5g), a strong reduction in sieve element formation outside of the primary phloem poles region (that is, de novo sieve element formation) was observed (Fig. 2j,k). The ratio of de novo sieve element numbers to vasculature diameter was significantly reduced in the triple mutant (Extended Data Fig. 5g), implying that the reduction in de novo secondary sieve element formation is not caused by general growth retardation. These findings suggest that *PAPL* genes are required for secondary conductive phloem formation. Collectively, these findings suggest that one of the functions of xylem and phloem parenchyma cells is to support conductive cell formation.

### Mature phloem parenchyma involved in biotic stress responses

**Fig. 3:**
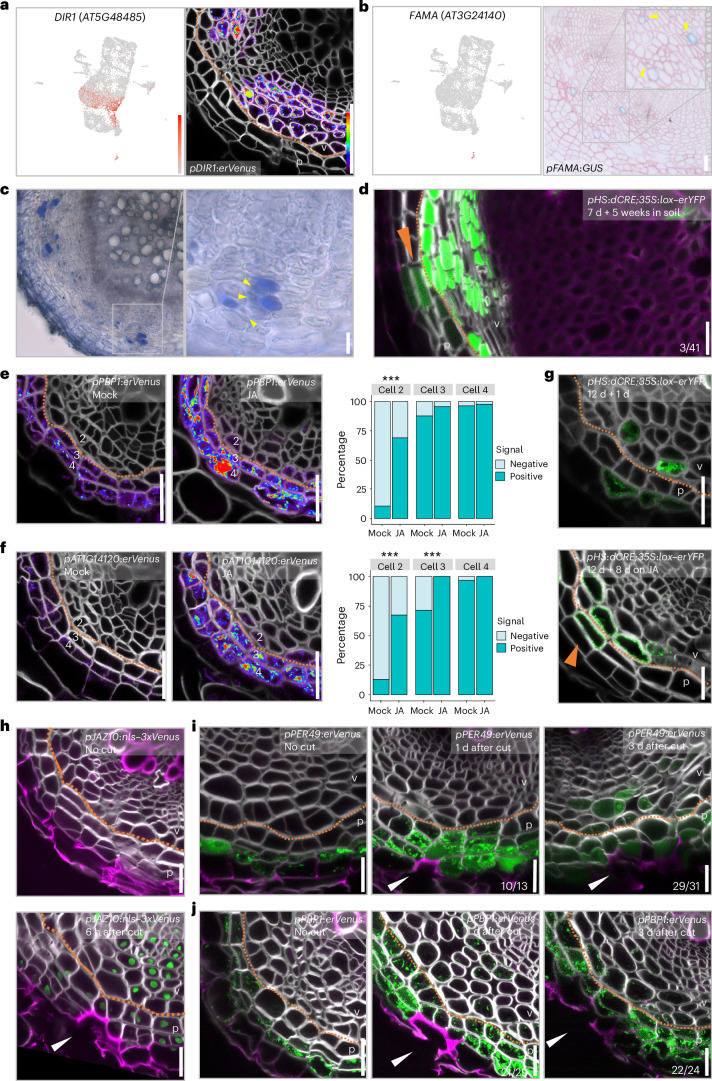
Mature phloem parenchyma functions in stress response and respecification of the barrier upon injury. **a**, UMAP plot and confocal cross-section of 16-day-old promoter–reporter line of *DIR1*, which is highly detected in mature phloem parenchyma clusters. **b**, UMAP plot and light microscopy image of a cross-section of the promoter–reporter line of *FAMA* specifically detected in the MI cluster. Detailed information on the reporters in **a** and **b** is available in Supplementary Table 4. **c**, Bright-field cross-section of a wild-type root with CBB staining. Panels **b** and **c** show cross-sections of 30-day-old roots. The yellow arrowheads point at sieve elements adjacent to the MIs. **d**, Confocal cross-section of *pHS*:*dCRE*;*35S*:*lox–erYFP* root. Clones induced in 7-day-old seedlings were analysed after 5 weeks of growth on soil. The fraction indicates that 3 out of 41 sectors extended to the periderm. **e**,**f**, Confocal cross-sections of 12-day-old *pPBP1*:*erVenus* (**e**) and *pAT1G14120*:*erVenus* (**f**) roots grown without (Mock) or with (JA) methyl jasmonate for 2 days, and the frequency of signal-positive cells in each cell in a cell file. The numbers in the cross-sections indicate cell file numbers in the bar charts. A two-sided Fisher’s exact test was performed, ***P < 0.001. *P*_*pPBP1*:*erVenus_cell2*_ = 8.411 × 10^−10^; *P*_*pAT1G14120*:*erVenus_cell2*_ = 3.11 × 10^−9^; *P*_*pAT1G14120*:*erVenus_cell3*_ = 2.68 × 10^−6^. **g**, Cross-sections of *pHS*:*dCRE*;*35S*:*lox–erYFP* roots. Clones induced in 12-day-old seedlings were analysed after 1 day of growth on half-strength Murashige and Skoog growth medium (1/2 GM) plates (top) and after 8 days of growth on 1/2 GM plates supplemented with 10 µM JA (bottom). In **d** and **g**, the orange arrowheads indicate the periderm sectors that have formed as a result of the transformation from phloem tissue. **h**, Confocal cross-sections of 17-day-old *pJAZ10*:*nls–3xVenus* roots without injury (top) or 6 hours after the phellem and phellogen were damaged (bottom). **i**,**j**, Confocal cross-sections of *pPER49*:*erVenus* (**i**) and *pPBP1*:*erVenus* (**j**) roots without injury (left) and 1 day (middle) or 3 days (right) after the phellem and phellogen were damaged in 17-day-old roots. In the cross-sections in **a** and **d**–**j**, cell walls were stained with SR2200. In **d** and **g**–**j**, green indicates YFP, and magenta indicates Basic Fuchsin. In **a** and **d**–**j**, the orange dotted line indicates the clonal boundary of vascular tissue and periderm. In **a**, **d** and **g**–**j**, v, vascular region; p, periderm. The relative expression levels of genes in UMAP and Venus signals in cross-sections are shown according to the colour scale in **a**. In **h**–**j**, the white arrowheads indicate the wounding site. The experiments in **c**–**j** were independently repeated three times. Scale bars, 20 µm (**a**–**j**). Source data

**Extended Data Fig. 6:**
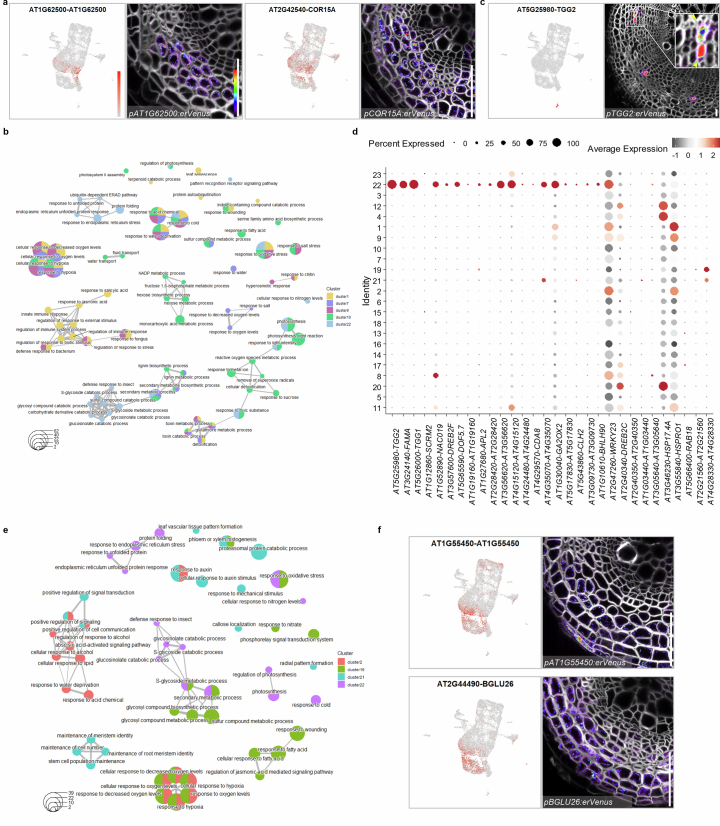
Analysis of mature phloem parenchyma clusters. **a**, UMAP plots of genes (*AT1G62500* and *COR15A*) highly detected in mature phloem parenchyma clusters and confocal cross-sections of their transcriptional reporter lines. **b**, GO enrichment analysis and comparison among DEGs of cluster 1, 7, 9, 10 and 22 shown with emapplot. The Top 30 enriched biological processes were shown. **c**, UMAP plot of *TGG2* specifically detected in MI cluster and confocal cross-section of its 30-day-old reporter line root. Yellow arrows point at the sieve elements adjacent to the MIs. **d**, Expression of potential MI differentiation regulators in our scRNA-seq data. Dot size represents the percentage of cells expressing a given gene; colour of the dot indicates the scaled average expression level. **e**, GO enrichment analysis and comparison among DEGs of cluster 2, 19, 21 and 22 shown with emapplot. The top 15 enriched biological processes were shown. **f**, UMAP plots of genes (*AT1G55450* and *BGLU26*) highly detected in part of mature phloem parenchyma clusters and periderm clusters and confocal cross-sections of their transcriptional reporter lines. Confocal cross-sections of **a**, **f** were from 16-day-old roots. In cross sections of **a**, **c**, **f**, cell walls stained with SR2200 were shown in grey. For each gene, the UMAP plot was shown in left and cross section was shown in right. Relative expression levels of genes in UMAP and Venus signals in cross sections were shown according to the color map in **a**. The detailed information of reporters is available in Supplementary Table 4. In **b** and **e**, dot size represents number of DEGs in given cluster involved in the biological process. The edge represents the similarity between terms and shorter and thicker edge indicates higher similarity. Circle highlights the different biological process of interest. The full list of GO terms of all the clusters is available in Supplementary Table 3. Scale bars, 20 µm (**a**, **c**, **f**).

As continuous growth pushes the more mature phloem parenchyma cells outward, these cells become larger and more loosely arranged than the young parenchyma cells, which are positioned near the vascular cambium. On the basis of this morphological characteristic and enriched marker expression in clusters 1, 7, 9 and 10, we named these large parenchyma cells ‘mature phloem parenchyma’ cells. This cell type is marked by the expression of, for example, *DEFECTIVE IN INDUCED RESISTANCE 1* (*DIR1*), *COLD-REGULATED 15A* (*COR15A*) and *AT1G62500* marker genes (Fig. 3a and Extended Data Fig. 6a). GO enrichment analysis of the mature phloem parenchyma clusters indicates that these cells are mainly involved in stress responses. Additionally, cells in cluster 10 appear to be involved in carbohydrate metabolic processes, indicating a potential storage function of mature phloem parenchyma cells (Extended Data Fig. 6b).

Through analysis of the top differentially expressed genes of cluster 22 and the reporter lines, we identified the presence of myrosin idioblasts (MIs) within the mature phloem parenchyma region (Fig. 3b,c and Extended Data Fig. 6c,d). MIs are specialized parenchymal storage cells that play a key role in the glucosinolate–myrosinase defence system, unique to Brassicaceae plants, which provides protection against herbivory^32–34^. The majority of known or predicted MI-related genes^35^ were expressed in this cluster (22 out of 30 genes; Extended Data Fig. 6d). Furthermore, signals of the myrosinase/thioglucosidase-encoding gene *THIOGLUCOSIDE GLUCOHYDROLASE2* (*TGG2*)^36^ and the MI regulator *FAMA*^34,35^ were sparsely detected in individual cells in the mature phloem parenchyma region (Fig. 3b and Extended Data Fig. 6c). We used Coomassie brilliant blue (CBB) staining to visualize the MIs^37^, and CBB-stained cells were scattered in a similar area where *TGG2*- and *FAMA*-positive cells were found (Fig. 3c). This demonstrates that MIs exist in the mature root. We often detected promoter activities of *TGG2* and *FAMA*, and CBB staining, in cells next to the sieve elements, the position occupied by the companion cells earlier in development (Fig. 3b,c and Extended Data Fig. 6c). From GO enrichment comparison among the conductive phloem and MI clusters (clusters 2, 19 and 21 and cluster 22, respectively), companion cells (cluster 19) seem to have a connection with MI cells in the context of glucosinolate catabolic and sulfur compound metabolic processes (Extended Data Fig. 6e). In summary, although we cannot exclude other origins, our data suggest that MI cells mature from companion cells in the phloem lineage.

### Injury transforms mature phloem parenchyma cells to periderm

**Extended Data Fig. 7:**
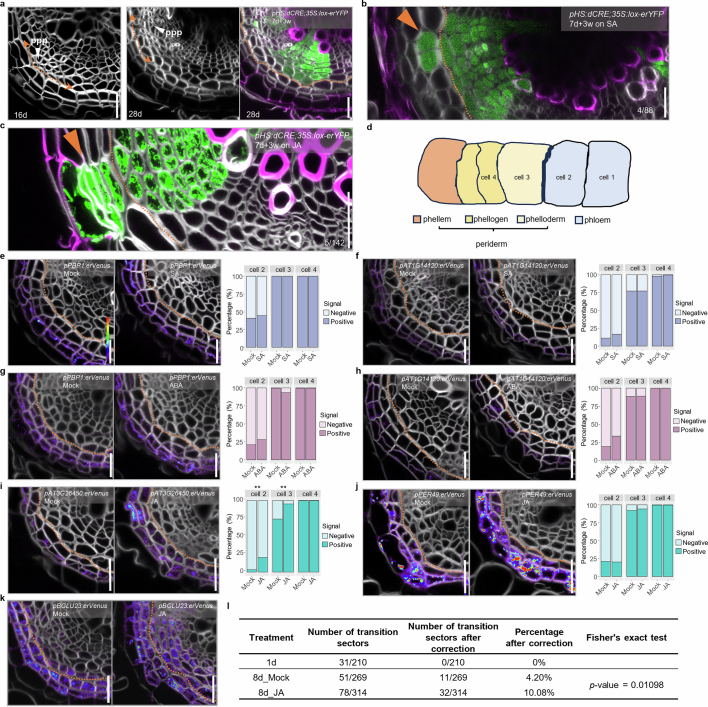
Obtaining periderm identity upon stress hormone treatment. **a**, Confocal cross-sections of 16-day-old and 28-day-old root. The rightmost panel showing a fluorescence sector originated from a single cell clone induced in procambium (*pHS:dCRE*;*35S:lox-erYFP)*. The boundary between the mature phloem parenchyma and periderm was determined based on the cell wall thickness and the boundary in the primary phloem pole (PPP) (detailed description in Method). **b**, **c**, Cross-section of *pHS:dCRE*;*35S:lox-erYFP* root. Clones induced in 7-day-old seedlings were analyzed 3 weeks after growth on 1/2GM plates supplemented with 10 µM SA (**b**) or 10 µM MeJA (**c**). The orange arrowheads indicate the periderm sectors that have formed as a result of the transformation from the phloem tissue. The fraction indicates 4 out of 88 sectors (**b**) extended to the phelloderm or 5 out of 142 sectors (**c**) showing two cell or one cell file invasion. In **a**-**c**, Green; YFP, magenta; Basic Fuchsin. Experiments of **a**-**c** were independently repeated three times. **d**, Schematic illustration of cell-number definition in a cell file for quantification. Cell 1 and 2 are mature phloem parenchyma cells and cell 3 and 4 are periderm cells. Cell 2 is the most distal mature phloem parenchyma cell next to the periderm and cell 1 is the parenchyma cell proximally adjacent to cell 2. Cell 3 is the most proximal phelloderm cells next to the mature phloem parenchyma and cell 4 is the periderm cell distally adjacent to cell 3. **e**, **f**, Confocal cross-sections of 12-day-old *pPBP1:erVenus* (**e**) and *pAT1G14120:erVenus* (**f**) root grown without (Mock) or with (SA) 10 µM SA for 2 days and the ratio of signal positive cells in each numbered-cell defined in **d**. **g**, **h**, Confocal cross-sections of 12-day-old *pPBP1:erVenus* (**g**) and *pAT1G14120:erVenus* (**h**) root grown without (Mock) or with (ABA) 10 µM ABA for 2 days and the ratio of signal positive cells in each numbered-cell defined in **d**. **i**, **j**, Confocal cross-sections of 12-day-old *pAT3G26450:erVenus* (**i**) and *pPER49:erVenus* (**j**) roots grown without (Mock) or with 10 µM MeJA (JA) for 2 days and the ratio of signal positive cells in each numbered-cell defined in **c**. In **e**-**j**, two-sided Fisher’s exact test was performed, **p < 0.01. *p*_*pAT3G26450_cell2*_ = 0.008381, *p*_*pAT3G26450_cell3*_ = 0.001808. **k**, Confocal cross-sections of 12-day-old *pBGLU23:erVenus* roots grown without (Mock) or with (JA) 10 µM MeJA for 2 days. **l**, Summary of 8 days lineage tracing analysis. The frequency of transition sectors in 1 day after heat shock was subtracted from those in 8 days. The numbers of transition sectors after the subtraction were rounded, two-sided Fisher’s exact test was performed. In cross sections of **a-c**, **e**-**k**, cell walls stained with SR2200 were shown in grey. The orange dotted lines and arrows indicate the boundary between mature phloem parenchyma and periderm. Scale bars, 20 µm (**a-c**, **e**-**k**). Source data

In our dataset, the periderm clusters 12 and 3 were adjacent to, but clearly separated from, mature phloem parenchyma clusters 1, 7, 9 and 10 (Fig. 1b). Reporter lines of genes highly expressed in clusters 3 and 12 exhibited specific fluorescence in the periderm (Fig. 1e and Extended Data Fig. 1f), while mature phloem parenchyma cluster markers were specifically detected in phloem parenchyma cells located near the periderm (Fig. 3a and Extended Data Fig. 6a). This separation is consistent with the observation that periderm and phloem parenchyma cells have different origins in primary tissue: the periderm originates from pericycle cells, and the phloem parenchyma primarily from procambium cells^3,11^. The clonal boundary is thus well defined, especially in young secondary tissues^11^ (Fig. 3a and Extended Data Fig. 7a). However, in addition to the periderm- or mature-phloem-parenchyma-specific genes (Figs. 1e and 3a and Extended Data Figs. 1f and 6a), we frequently found genes that were detected in both mature phloem parenchyma and periderm clusters, and their reporter lines validated expression in both tissues (Extended Data Fig. 6f). Moreover, in the UMAP, there was a small group of cells between mature phloem parenchyma and periderm clusters (Fig. 1b). Since the phelloderm is adjacent to the mature phloem parenchyma in secondary tissues, we hypothesized that mature phloem parenchyma cells gradually obtain phelloderm identity. To test this hypothesis, we carried out lineage tracing by inducing YFP clones in procambial cells within a few days after secondary growth activation. Five weeks after induction, we examined non-xylem-pole-pericycle lineage sectors in which phloem parenchyma and periderm have distinct origins^11^. We found that the majority of these sectors reached the boundary of the periderm and mature phloem parenchyma (35 out of 41 sectors). However, a small proportion of sectors extended into the periderm. We found single phelloderm cells (one-cell invasion) (3 out of 41 sectors) or even an entire radial periderm cell file (one-cell-file invasion) (3 out of 41 sectors) that originated from the procambium (Fig. 3d). These data show that mature phloem parenchyma cells have the capacity to obtain periderm identity, albeit at low frequency under normal growth conditions.

We next investigated the biological role of this transformation. Mature phloem parenchyma clusters showing a gradual transformation to the periderm (clusters 1 and 9) were overrepresented by jasmonic acid (JA) and salicylic acid (SA) responses and response to biotic stress (Extended Data Fig. 6b). These data suggest that mature phloem parenchyma cells might be preparing for biotic stress, and these stress hormones could promote the transformation of the phloem parenchyma to the periderm to reinforce the root barrier. To examine the potential role of JA and SA in this transformation, we performed a 3-week lineage tracing experiment upon JA or SA treatment (Supplementary Notes and Extended Data Fig. 7b,c). The results show that treatment with JA and SA accelerated the transformation of mature phloem parenchyma cells to periderm cells. This accelerated transformation seems not to be caused by general growth retardation caused by JA and SA^38,39^, since abscisic acid (ABA), another stress hormone and growth inhibitor^40^, did not accelerate the transition, despite being able to slow down radial growth (Supplementary Notes). We wondered whether such a hormone treatment might affect cell identities in the mature phloem parenchyma cells as a part of the transition process. Hormone treatment was first applied to two periderm marker lines, *PBP1* and *AT1G14120*. JA treatment resulted in the most significant expansion of periderm marker expression inward, into mature phloem parenchyma (Fig. 3e,f). SA or ABA treatment, however, showed a minor effect on these periderm markers (Extended Data Fig. 7d–h). We also tested JA treatment with other periderm markers generated from this study. *AT3G26450* showed clear signal expansion, and *PER49* showed slight expansion in reporter expression upon JA treatment. The reporter line of *BGLU23*, which is known to be JA inducible^41^, showed drastic signal increase and expansion in the whole root after JA treatment (Extended Data Fig. 7i–k). The periderm marker expression showed its expansion after JA treatment in 2 days, suggesting that the transformation of phloem into periderm could occur more rapidly than in 3 weeks (Fig. 3e,f). Accordingly, we observed a clear increase in clone invasion by JA as early as after 8 days of lineage tracing (Fig. 3g, Supplementary Notes and Extended Data Fig. 7l; 4.20% transition rate with Mock, 10.08% with JA). With our lineage tracing method, we can confidently trace only recent transition events (Supplementary Notes). Since 8 days of JA treatment resulted in a higher frequency of transition events than 3 weeks of treatment, our data suggest that the majority of the JA-induced transition events occur during the first days after JA application.

Given that JA signalling is induced upon tissue injury^42^, we hypothesized that the transition is beneficial for reinforcing the barrier upon superficial injury caused by, for example, growth in soil^42^. To mimic superficial injury, we ablated the phellem and phellogen with a shallow longitudinal cut along mature roots with a razor blade. We confirmed that superficial injury was sufficient to induce the expression of JA response marker *pJAZ10*:*nls–3xVenus* near and further away from the cut site 6 hours after the injury (Fig. 3h). One day after the injury, the periderm markers, *PER49* and *PBP1*, expanded their expression into the mature phloem parenchyma cells, beneath the wound (Fig. 3i,j). These observations suggest an inward identity shift after the superficial injury. The re-establishment of phellogen in former phelloderm cells and the phellem cells adjacent to them was first observed 3 days after injury. *PER49* and *PBP1* expression were maintained beneath the wound site to mark the newly formed periderm (Fig. 3i,j). Collectively, our data indicate that the phelloderm and phloem parenchyma function as a reservoir for the phellogen and thus for barrier re-establishment. During normal development, the transformation from phloem parenchyma via phelloderm to phellogen is slow and sporadic, but this can be accelerated to reinforce the barrier in case of superficial injury.

## Discussion

**Fig. 4:**
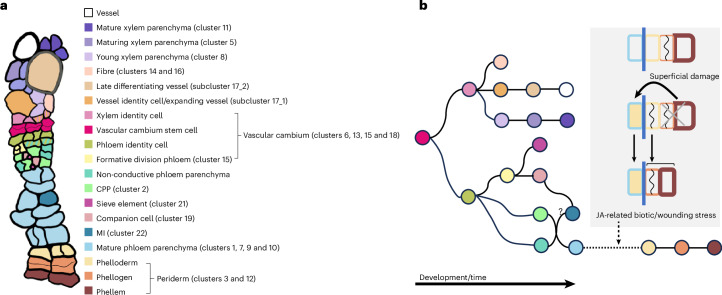
A hierarchical cell fate determination map of the *Arabidopsis* root undergoing secondary growth. **a**, Cell types in the *Arabidopsis* root secondary tissues. Cell type classification was based on previous reports and data presented in this study. The cluster IDs correspond to the cluster IDs in the UMAP. **b**, Hierarchical cell fate determination in secondary tissue. The circle colours represent the cell identities in **a**.

**Extended Data Fig. 8:**
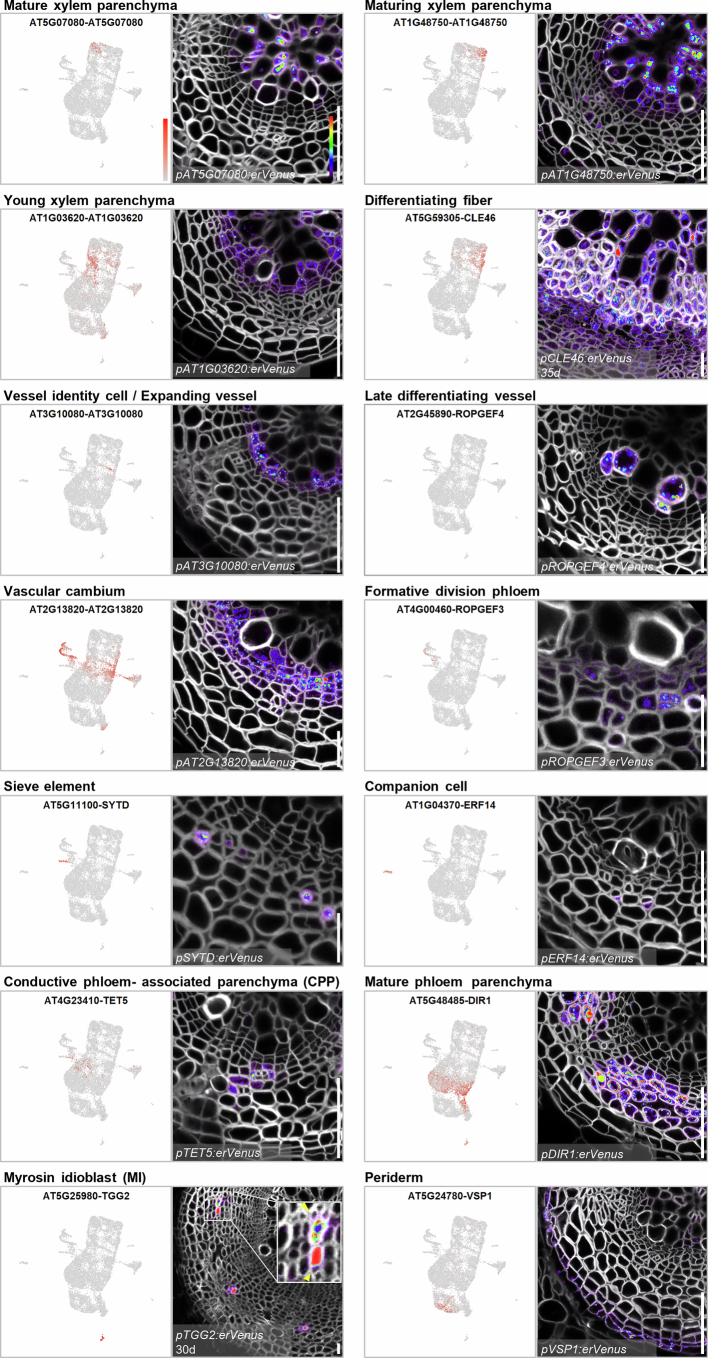
Representative markers for each cell type in the mature root of Arabidopsis thaliana. Relative expression levels of genes in UMAP and fluorescence signals in cross sections were shown according to the color map. Cell walls stained with SR2200 were shown in grey. The detailed information of reporters is available in Supplementary Table 4. Scale bars, 20 µm.

**Extended Data Fig. 9:**
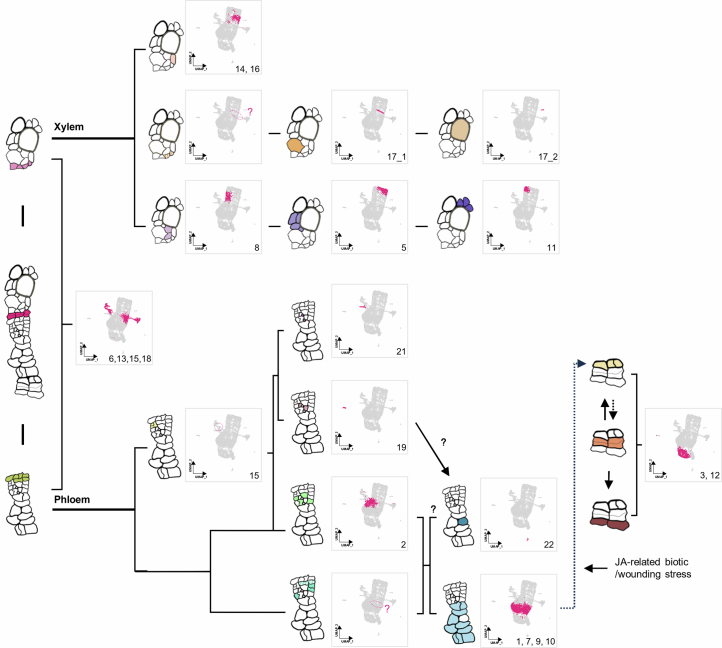
A detailed hierarchical cell fate determination map of the Arabidopsis root undergoing secondary growth. A detailed schematic illustrates hierarchical cell fate determination in root secondary tissue. The cross-sections on the left highlight cell types, with colors indicating their identities as shown in Fig. 4a. On the right, UMAPs display the corresponding clusters, with cluster IDs noted at the bottom right corner. Red circles mark cells with potentially shared identities that were not separated by clustering.

In this study, we used scRNA-seq and extensive reporter analysis to identify cell types and states in the *Arabidopsis* root undergoing secondary growth (Fig. 4a and Extended Data Fig. 8). By combining prior knowledge and our findings, we were able to generate a hierarchal cell fate determination map for all cell types during secondary growth in roots (Fig. 4b and Extended Data Fig. 9). The vascular cambial stem cells produce xylem cells inward and phloem cells outward^1,3,11,43^. Our data indicate that xylem-side stem cell daughters first obtain common xylem identity before specifying into one of three different cell types: vessels, parenchyma or fibre cells. Like the visibly differentiated xylem cell types, we found that the xylem parenchyma also undergoes several maturation steps. The phloem-side stem cell daughters obtain phloem identity before becoming phloem parenchyma cells, or they undergo formative divisions to form conductive phloem cells (sieve elements and companion cells) and CPP cells (Fig. 4b). We discovered that CPP cells are also recruited from the parenchyma cell lineage by the adjacent conductive phloem cells. As vascular cambium produces more phloem cells, the previously produced phloem parenchyma cells enlarge and become mature phloem parenchyma cells. MI cells are also developed within the mature phloem parenchyma region, possibly by differentiating from mature companion cells.

We unexpectedly found that the mature phloem parenchyma cells gradually change their identity into phelloderm cells, the innermost cell type of the periderm. Our data suggest that upon physical damage of the periderm, JA signalling is triggered, which in turn stimulates the accelerated transformation of mature phloem parenchyma into periderm cells to quickly reinforce the barrier. Since wounding caused more frequent cell transformation than JA treatment, we hypothesize that there are other factors than JA that promote the transformation. It has been reported that damage-induced JA signalling in root apical meristem triggers stem cell regeneration to sustain primary root growth^44^. The underlying mechanism for the transformation of phloem parenchyma into the periderm is unknown, but it is possible that the common regulatory factors are used for stem cell regeneration in the root tip and periderm replenishment. In addition, biotic stresses, such as bacteria, fungi or herbivore attack, can also cause damage to the periderm and activate JA-induced barrier reinforcement. Moreover, herbivore attack can cause deeper damage to the secondary tissue, which can be confronted with the MI cells located within the mature phloem parenchyma. Breakage of the MI cells and the neighbouring mature phloem parenchyma cells in Brassicaceae such as *Arabidopsis thaliana* triggers an herbivory defence response called the mustard oil bomb^33,34^. Thus, two elaborate defence mechanisms are elicited upon injury depending on the depth of damage to protect the mature root. Protection of the mature root is critical for the plant’s survival, as it connects the whole root system to the entire shoot system above.

Secondary tissues of tree species are more complex than those of the model species *A. thaliana*. In addition to the morphologically similar parenchyma cell types and cell states we identified in this study, tree species have morphologically distinct parenchyma cell types, such as ray cells^1^. A few single-cell and single-nuclei transcriptome analyses have been published recently^45,46^, but extensive verification of these datasets with independent methods is needed to be able to discover the full spectrum of cell types in tree secondary tissue.

Storage organs consist of specialized secondary tissue produced by vascular cambium of several important crop species, such as cassava and sweet potato^47,48^. A majority of tissues in storage organs are composed of xylem parenchyma cells, which store carbohydrates and other nutrients^49^. While *Arabidopsis* secondary tissue appears to lack specific storage parenchyma cell types, our parenchyma transcriptome dataset will be valuable in identifying state-specific roles of parenchyma cells, which may have a function in corresponding storage cell types in crop species.

## Methods

### Plant growth conditions

The seeds were sterilized with 20% chlorine for 3 min followed by sterilization with 70% ethanol for 5 min and were washed with Milli-Q water twice. The seeds were kept in the dark at 4 °C for 2 days and then plated on 1/2 GM supplemented with 2.2 g l^−1^ MS salt mixture with vitamins (Duchefa), 1% sucrose, 0.5 g l^−1^ MES (Duchefa) and 0.8% agar (Duchefa) (pH 5.8). The T_1_ generation seeds were mixed with 0.1% agar supplemented with 250 µg ml^−1^ cefotaxime before plating to avoid *Agrobacterium* contamination. The plates were placed vertically in a growth chamber. The day when the plates were moved to the growth chamber was defined as day 0. Analysis was performed with plate-grown seedlings unless otherwise mentioned. The seedlings were transferred to soil around day 7 if necessary for analysis. The plants were grown at 23 °C under long-day photoperiod conditions (16 hours light/8 hours dark).

### Protoplast isolation and florescence-activated cell sorting

We used 30-day-old *pPXY*:*erYFP* (Col-0) grown on soil to isolate protoplasts for single-cell analysis. At this age under our growth conditions, these seedlings had just initiated flowering, which has been shown to trigger the transition from phase I to II of secondary development^8,50^. In this way, we were able to obtain all the cell types during secondary growth, including cells developing into each of the three xylem cell types (vessels, parenchyma and fibres). The protoplast isolation protocol was modified from the published protocol^51^. Roots within 2 cm below the root–hypocotyl junction were harvested (lateral roots were removed) and washed with tap water and then Milli-Q water. The roots were longitudinally dissected with a razor blade under a stereo microscope and put into protoplast isolation solution (1.5% (w/v) cellulase-R10 (Yakult), 0.4% (w/v) macerozyme-R10 (Yakult), 0.4 M mannitol (Sigma-Aldrich), 20 mM MES (Duchefa), 20 mM KCl (1 M stock in Milli-Q water), 0.1% (w/v) BSA (Sigma-Aldrich) and 10 mM CaCl_2_ (1 M stock in Milli-Q water)). The samples were incubated at room temperature under dark conditions with gentle shaking (75 rpm) for 1 hour. After the incubation, the solution was filtered once with a 70-µm cell strainer, and the flow-through was centrifuged at 400 *g* for 6 min. The supernatant was gently removed, and the protoplasts were resuspended in the buffer (the protoplast isolation solution without enzymes). The resuspended protoplast solution was filtered three times with a 40-µm cell strainer. Protoplasts were stained with 14 µM 4′,6-diamidino-2-phenylindole (DAPI) in phosphate-buffered saline (PBS), and the DAPI-negative cells were sorted using BD FACSAria II. We confirmed the efficiency of this protocol by isolating protoplasts from known tissue-specific reporters covering the major cell types in the root secondary tissue. We were able to capture fluorescence-positive protoplasts from each reporter line, suggesting that our protoplast isolation method was sufficient to acquire cells residing in different regions of the secondary tissue.

### Single-cell RNA-seq sample processing, library establishment and sequencing

After sorting, the protoplasts were centrifuged at 400 *g* at 4 °C for 5 min and then resuspended in resuspension solution to a final concentration of around 1,000 cells per µl. The resuspended cells were loaded on a Chromium Single Cell 3′ GEM, Library & Gel Bead Kit (V3 chemistry, 10X Genomics) according to the manufacturer’s instructions. Libraries were sequenced on an Illumina HiSeq4000 and NovaSeq6000 instrument following the recommendations of 10X Genomics at the VIB Nucleomics Core facility (VIB, Leuven).

### Processing of raw sequencing data and data analysis

The FASTQ files obtained after demultiplexing were used as the input for cellranger count (v.6.1.2), and reads were mapped to the *A. thaliana* reference genome (Ensembl TAIR10.40). Initial filtering in cellranger recovered 17,140 cells, corresponding with a mean of 33,679 reads per cell and a median of 2,409 genes per cell. Further data processing was performed in R (v. >3.6.0) (https://www.r-project.org/) using the scater package^52^ (v.1.10.1). Outlier cells were defined as having less than 4,000 UMIs or as cells containing more than 5% mitochondrial or chloroplast transcripts. After outliers were removed, 11,760 cells were retained for further analysis. Normalizing the raw counts, detecting highly variable genes, finding clusters and creating UMAP plots were done using the Seurat package (v.4.1.0)^53^. Differential expression analysis for marker gene identification per subpopulation was based on the non-parametric Wilcoxon rank sum test implemented within the Seurat pipeline. The necessary reported information to allow the evaluation and repetition of a plant single-cell/nucleus experiment is included in Supplementary Table 1.

### GO enrichment analysis

The differentially expressed gene list (average log_2_(fold change) ≥ 0.5) for each cluster or subcluster was used for GO enrichment analysis using clusterProfiler v.4.2.2 (ref. ^54^) in the program R v.4.0.2 (https://www.r-project.org/) with *P* < 0.05.

### Gene expression images with the Cella model

The Cella model of mature root was extracted from a 30-day-old mature root cross-section, which was used as a template in Adobe Photoshop 2024 (https://www.adobe.com/products/photoshop.html). The image was made black and white. The 3D modelling manual protocol^26^ was then followed in Blender v.4.0.1 (https://www.blender.org/download/releases/4-0/) until cell extrusion and continued with model annotation and image output with averaged cluster data. The R export code, blend file, Python scripts and input Excel sheets and all the gene expression results have been deposited in figshare (10.6084/m9.figshare.27569697.v2). The aesthetics of the Cella output images (Fig. 1d,f) are different from the database versions at https://single-cell.be/plant/root-secondary-tissue due to technical reasons.

### Cloning of reporter lines and plant transformation

We selected genes for cluster validation on the basis of their expression in the UMAP. Their promoter regions were amplified with the primers listed in Supplementary Table 4 and cloned into the *pDONRP41R* entry vector as the first box using Gateway BP Clonase II enzyme (Thermo Fisher Scientific). The first box plasmid (promoter); the secondary box plasmid (reporter gene), *221z–erVen* or *221z–erRFP*^55^; the third box plasmid (terminator), *2R3e–3AT*^55^; and the destination vector, *pFRm43GW*^56^ containing an *RFP* seed coat selection marker, were used to generate the reporter line constructs via MultiSite Gateway technology (Thermo Fisher Scientific). The genes selected for the reporter analysis, the primers used for promoter amplification, the frequency of T_1_ individuals showing the expression pattern predicted from the scRNA-seq dataset and the available generation of the reporter seeds are listed in Supplementary Table 4. The generated promoter:*erVenus*–*YFP* constructs were transformed into Col-0, and the reporter expression was examined in the T_1_ generation unless otherwise stated. The *pTET5*:*erRFP* construct was transformed into *pHS*:*dCRE;p35S*:*lox–erYFP*.

### Mutant analysis

In this study, Col-0 was used as the wild type. The *3paplC* triple mutant has been described previously^20^. The rest of the mutants were ordered from the Nottingham Arabidopsis Stock Centre: *apk1a* (GK-430G06), *at4g30460* (SALK_140721), *anac087* (SALK_079821), *myb3r-3* (SALK_143357C), *myb3r-5* (SALK_205058C), *nac055* (SALK_014331C), *myb112* (SALK_017020C), *anac046* (SALK_107861C) and *at4g21620* (SALK_099390C). The mutants ordered from the Nottingham Arabidopsis Stock Centre were genotyped, and homozygous lines were selected for seed propagation. All the mutants and Col-0 were freshly propagated for phenotyping,

The mutant phenotypes were examined using 14-day-old seedlings except for *3paplC*, which were 16 days old. The phenotyping of *3paplC* and the other mutants was repeated four times or twice, respectively.

### Lineage tracing analysis

For lineage tracing analysis, we generated transgenic plants in which erYFP (YFP targeted to the endoplasmic reticulum) sectors can be activated upon heat shock using a previously developed lineage tracing system based on the d-Box-CRE (hereafter dCRE) fusion protein^11^. We backcrossed the *pHS*:*dCRE;35S*:*lox–GUS* line with Col-0 and established the F_3_ line, which was homozygous for *pHS*:*dCRE* without the *35S*:*lox–GUS* construct (*pHS*:*dCRE*) on the basis of selection markers (hygromycin for *pHS*:*dCRE* and Basta for *35S*:*lox–GUS*). The *35S*:*lox–erYFP* construct was transformed into the *pHS*:*dCRE* seedlings; the T_3_ lines homozygous for the *35S*:*lox–erYFP* construct (*pHS*:*dCRE;35S*:*lox–erYFP*) were established on the basis of the selection marker (Basta). T_3_ or T_4_ seedlings were used for analysis. *pTET5*:*erRFP* was transformed into *pHS*:*dCRE;35S*:*lox–erYFP* and was analysed at the T_2_ generation.

For clone induction, 7-day-old or 12-day-old seedlings grown on 1/2 GM plates were used. After excess water was removed from the plates, one or two plates were placed in a plastic bag. We sealed the plastic bag with plastic tape to avoid water leakage and submerged the bag in 37 °C water for 20 min. This temperature is typically encountered by plants during the summer and does not induce significant stress. We retrieved the plates from the plastic bag and kept them horizontally at 4 °C for 30 min. The plates were then moved to the growth chamber. One day later, the sector induction was examined under a fluorescence stereo microscope. For sector analysis at 5 weeks after the induction, the seedlings were moved to soil. For sector analysis with hormone treatment, the seedlings were transferred to 1/2 GM plates supplemented with hormone.

### Chemical treatment

For hormone treatment, SA (Sigma-Aldrich), methyl jasmonate (JA, Sigma-Aldrich) and ABA (Duchefa) were dissolved in DMSO (SA and JA) or ethanol (ABA) to prepare 100 mM stock solution. The stock solution was stored at −20 °C. To examine gene expression upon treatment, the homozygous T_3_ reporter lines were first grown on 1/2 GM plates for 10 days and then transferred to 1/2 GM plates supplemented with 5 µM SA, 10 µM JA or 10 µM ABA, or 1/2 GM plates containing an equal volume of DMSO or ethanol as a control (called Mock in the experiments). The seedlings were treated for 2 days.

For lineage tracing analysis with hormone treatment, 7-day-old seedlings grown on 1/2 GM plates were used to induce clones upon heat shock as described above. One day later, the seedlings were transferred to 1/2 GM plates supplemented with 5 µM SA, 10 µM JA or 10 µM ABA, or 1/2 GM plates containing an equal volume of DMSO or ethanol as a control (Mock). The seedlings were grown for 20 days.

We prepared 17-β-oestradiol (EST, a synthetic derivative of oestrogen (Sigma-Aldrich)) as 20 mM stock in DMSO and stored the stock solution at −20 °C. The 13-day-old *35S*:*XVE*»*CKX7–RFP*^57^ seedlings were transferred to 1/2 GM plates containing 5 µM EST or an equal amount of DMSO (Mock) for 1 day.

### Fluorescence marker fixation, vibratome sectioning and confocal imaging

The protocol was modified from a previously published paper^11^. To examine fluorescence reporter expression, samples were fixed in 4% paraformaldehyde (Sigma-Aldrich) in 1×PBS (pH 7.2) supplemented with 0.1% triton under vacuum for 1 hour, and then washed twice with 1×PBS. The roots were embedded into 5% agarose (Sigma-Aldrich) in 1×PBS, followed by vibratome sectioning. The 200-µm-thick slices were kept in 1×PBS supplemented with 1 µl ml^−1^ Renaissance SCRI 2200 (SR2200) (Renaissance Chemicals) to stain the cell walls. To stain lignin, the agarose slices were put into ClearSee^58^ supplemented with 1 µl ml^−1^ SR2200 and 50 µg ml^−1^ Basic Fuchsin (Sigma-Aldrich)^59^. The cross-sections were mounted with 1×PBS or ClearSee and imaged with a Leica SP8 Stellaris confocal microscope (Leica) under a ×20 or ×63 objective with Leica Las AF software. All the confocal images were taken with sequential scan mode. For *35S*:*XVE*»*CKX7–RFP*^57^, the sections were stained with 1 μg ml^−1^ calcofluor white (Sigma-Aldrich) in 1×PBS. To visualize reporter gene expression, the settings were adjusted individually for each reporter line. However, when fluorescence signal intensities were compared within an experiment, identical settings were used. For the setting to visualize cell wall staining, signals were adjusted for each cross-section individually.

### GUS staining, material fixation, microtome sectioning and light microscopy

The GUS staining protocol was modified from a previously published paper^60^. Samples were kept in 90% acetone on ice for 30 min. After incubation, the samples were washed twice with 0.05 M sodium phosphate buffer (pH 7.2). The samples were then submerged in GUS solution (0.05 M sodium phosphate buffer (pH 7.2), 1.5 mM ferrocyanide, 1.5 mM ferricyanide, 1 mM X-glucuronic acid and 0.1% Triton X-100) and kept at room temperature under vacuum for 1 h. The samples were incubated at 37 °C until the desired GUS signals were detected.

Samples for microtome sectioning were fixed in 1% glutaraldehyde and 4% formaldehyde in 0.05 M sodium phosphate (pH 7.2) overnight and washed with 0.05 M sodium phosphate twice. To gradually dehydrate, the samples were kept in 10%, 30%, 50%, 70%, 96% and 100% ethanol for 30 min at each step. The 30 min incubation with 100% ethanol was repeated one more time. The samples were transferred to a 1:1 (v/v) solution of 100% ethanol and solution A (Leica Historesin Embedding kit) and kept for 1 hour, followed by incubation in solution A overnight. The samples were aligned in chambers and embedded with a 14:1 (v/v) solution of solution A and the hardener.

Cross-sections of the embedded samples were made with a Leica JUNG RM2055 microtome. The sections were acquired around 0.5 cm below the root–hypocotyl junction. The thickness of the sections was 10 µm for GUS reporter lines or otherwise 5 µm. The cross-sections of GUS reporter lines were stained with 0.05% (w/v) ruthenium red (Sigma-Aldrich); the cross-sections without GUS signals were additionally stained with 0.05% (w/v) toluidine blue (Sigma-Aldrich). The sections were imaged with a Leica 2500 microscope under ×20 and ×40 objectives.

### CBB staining

The protocol for CBB staining was modified from a previously published paper^37^. Thirty-day-old plants were collected in 15-ml CELLSTAR tubes (Greiner Bio-One) with CBB solution (45% methanol (Sigma-Aldrich), 10% acetic acid (Sigma-Aldrich) and 0.25% CBB R250 (Sigma-Aldrich)) and boiled in a water bath for 3 min, followed by washing with 1×PBS three times. The roots within 1 cm below the root–hypocotyl junction were embedded in 5% agarose and sectioned by vibratome at 100-µm thickness. The sections were mounted with water and imaged with a Leica 2500 microscope.

### Wounding experiment

We used 17-day-old seedlings for the wounding experiment. Roots within 5 mm of the root–hypocotyl junction were longitudinally injured with a razor blade under a dissection stereo microscope. We defined a superficial cut as the ablation of the phellem and phellogen. Only cross-sections with superficial cuts were used for analysis.

### Quantification and statistical analysis

For mutant phenotype quantification, the number of secondary vessels and sieve elements, vasculature diameter and cellular fluorescence signal intensity were manually measured using Fiji ImageJ v.1.52 (ref. ^61^). Phloem sieve elements were characterized using toluidine blue and ruthenium red staining. Ruthenium red stains pectin^62^, which is present in the cell walls of all plant cells. Toluidine blue differentially stains lignified tissues, nuclei and various cellular components, resulting in distinct colour variations depending on the chemical composition and structure of the cells^63,64^. Fully differentiated sieve elements are small and are not stained with toluidine blue. In contrast, adjacent companion cells are smaller and exhibit a darker stain with toluidine blue than neighbouring parenchyma cells.

To detect the transformation of mature phloem parenchyma into periderm, we detected the clonal boundary between the mature phloem parenchyma and periderm on the basis of thick primary cell walls between them. Since primary phloem poles and periderm are adjacent, even in mature root, we can use this position as an anchor to define the boundary in the neighbouring cell files (Extended Data Fig. 7a). Thus, on the basis of the boundary in the primary phloem region, cell wall thickness and cell arrangement, we could also confirm the boundary in the other regions in most cases. When the boundary is unclear (especially in cases when the transformation occurs in a radial cell file), we can use the clearly visible boundary of untransformed cell file as a ruler to detect transformation in the adjacent cell file.

For 3-week lineage tracing quantification, we considered only small-sector invasions into the periderm for the analysis (such as Fig. 3d), since we could not be sure whether the large-sector invasions were the result of a single recombination event or two separate recombination events in adjacent cells located on both sides of the clonal boundary. Thus, especially in the 3-week tracing experiments, we excluded clones that were initiated at the beginning of JA treatment. For 8-day lineage tracing quantification, to eliminate the possibility of counting sectors from two distinct single-cell clones across a boundary, we measured the frequency of this event 1 day after heat shock (31 out of 210 sectors; 14.76%). This frequency was subtracted from that observed in 8-day Mock (51 out of 269 sectors; 18.96%) or JA treatment (78 out of 314 sectors; 24.84%) samples. The numbers of transition sectors after correction were rounded (Mock, 11 out of 269 sectors, 4.20%; JA, 32 out of 314 sectors, 10.08%) (Extended Data Fig. 7l). See also Supplementary Notes.

All the plots for visualizing the quantification results were produced using the ggplot2 package (v.3.4.2)^65^ in RStudio (https://www.rstudio.com/) with the program R v.4.0.2 (https://www.r-project.org/). For the box plots, the boxes in represent the median values and interquartile range, and the whiskers indicate the total range. The black dots indicate measurements from individual roots. For each reporter line, after quantifying the cellular fluorescence signal intensity value of cell 1 to cell 4 (Extended Data Fig. 7d), cells in cells 2, 3 and 4 whose signal intensities were higher than the maximum signal intensity in cell 1 of Mock-treated roots were defined as signal-positive cells. The proportion of signal-positive cells from cell 2 to cell 4 was visualized using bar charts.

For mutant phenotype analysis, the normality distributions of each type of quantification were tested with the Shapiro–Wilk normality test. Significant differences were examined using the two-sided Wilcoxon test, except for the number of sieve elements, for which we used the two-sided *t*-test. For fluorescence signal analysis, the two-sided Fisher’s exact test was performed.

### Reporting summary

Further information on research design is available in the Nature Portfolio Reporting Summary linked to this article.

## Supplementary information

Supplementary Information Supplementary Notes and captions for Supplementary Tables 1–4.

Reporting Summary

Supplementary Table 1 Necessary reported information to allow evaluation and repetition of a plant single-cell/nucleus experiment.

Supplementary Table 2 Top differentially expressed gene list (DEGs) for each cluster or cluster combination versus the rest of the cells.

Supplementary Table 3 GO analysis of the top differentially expressed gene in each cluster.

Supplementary Table 4 Reporter summary.

## Source data

Source Data Fig. 2 Statistical source data.

Source Data Fig. 3 Statistical source data.

Source Data Extended Data Fig. 4 Statistical source data.

Source Data Extended Data Fig. 5 Statistical source data.

Source Data Extended Data Fig. 7 Statistical source data.

## Data Availability

The transcriptome data have been deposited in the online tool, and the raw data can be accessed at NCBI with GEO number GSE270140. Reads were mapped to the *A. thaliana* reference genome (Ensembl TAIR10.40). The data can be accessed via a freely accessible online browser tool (https://single-cell.be/plant/root-secondary-tissue). The input Excel sheets and gene expression results obtained with the Cella model are available via figshare at 10.6084/m9.figshare.27569697.v2 (ref. ^66^). Source data are provided with this paper. All other data are either in the main paper or Supplementary Information. Material requests should be directed to the corresponding authors.

## Code availability

For Cella model, the R export code, blend file and Python scripts are available via figshare at 10.6084/m9.figshare.27569697.v2 (ref. ^66^).
