# Supplementary material for: The dynamic and diverse nature of parenchyma cells in the Arabidopsis root during secondary growth
Source: Nat Plants. 2025 Mar 26;11(4):878–90. doi: 10.1038/s41477-025-01938-6 (PMC12014502; doi:10.1038/s41477-025-01938-6)
Supplement: Supplementary file 1 — Supplementary Notes and captions for Supplementary Tables 1–4. [file 41477_2025_1938_MOESM1_ESM.pdf]

nature plants

Supplementary information https://doi.org/10.1038/s41477-025-01938-6

The dynamic and diverse nature of parenchyma cells in the *Arabidopsis* root during secondary growth

In the format provided by the authors and unedited

Supplementary Notes

Identification of vascular cambium and periderm cells in the scRNA-seq data

First, we aimed to identify the clusters representing the vascular cambium and cork cambium. We reasoned that these cell types are presented in clusters dominated by cell cycle genes. To investigate this, we initially analysed the expression of core cell cycle genes[1] in our dataset. Cluster 15 and 18 were enriched in cells in the G2/M phase, and cluster 13 enriched in cells in the S phase. Because these cell-cycle gene expressions were also detected in part of clusters 2 and 6 close to clusters 13 and 15, these cells would also be undergoing cell divisions, forming a 'meristematic cell band' in the middle of the UMAP (Extended Data Fig. 1c). Gene Ontology (GO) comparison among differentially expressed genes (DEGs) of clusters 2, 6, 13, 15 and 18 showed cells in cluster 6, 13, 15 and 18 were involved in cell division and pattern specification, while cells in cluster 2 were involved in chemical and hormonal response (Extended Data Fig. 2), suggesting meristematic cells mainly exist in cluster 6, 13, 15, 18 and partially in cluster 2. Supporting this, the recently discovered stem cell factors of vascular cambium, *AINTEGUMENTA* and *PLETHORA* 5[2] are predominantly expressed in the cells representing the meristematic cell band (Extended Data Fig. 1b). We selected genes preferentially expressed in the meristematic cell band and examined their expression by generating transcriptional reporter lines. Consistently, promoter activities of *AT2G47780*, *AT2G13820*and *THAUMATIN-LIKE PROTEIN-1* (*ATLP-1*) were specifically and *RECEPTOR FOR ACTIVATED C KINASE 1C* (*RACK1C*), *INDOLE-3-ACETIC ACID 12* (*IAA12*) and *FANTASTIC FOUR 3* (*FAF3*) were preferentially detected in the vascular cambium (Fig. 1c and Extended Data Fig. 1e). Thus, the meristematic cell band clusters (6, 13, 15, 18 and subset of 2) contain the vascular cambium.

Periderm gene, *PEROXIDASE 15* (*PER15*)[3] is expressed in cluster 3 and 12, and a small group of dividing cells in clusters 13 and 18 (Extended Data Fig. 1b), genes with similar expression pattern (*PYK10-BINDING PROTEIN1* (*PBP1*), *PEROXIDASE 49* (*PER49*), *BETA-GLUCOSIDASE 23* (*BGLU23*), *AT3G26450*, *AT1G14120* and *VEGETATIVE STORAGE PROTEIN 1* (*VSP1*)) showed the expression of reporter gene in the periderm with different tissue preferences (Fig. 1e and Extended Data Fig. 1f). Fluorescence signals in *PBP1* and *BGLU23* reporter lines were detected in the entire periderm; *AT3G26450* and *VSP1* reporter lines showed the highest fluorescence signal intensities in the periderm cells with recently formed cell wall; reporter lines of *PER49* and *AT1G14120* were preferentially expressed in the phellem and phellogen and *PER49* expression peaked in the phellem (Fig. 1e and Extended Data Fig. 1f). The periderm cell types were not separated well between different clusters. *PER49*, *PER15* and *AT1G14120* predominantly expressed in the phellem tended to be detected in cells at the lower side of cluster 3 and 12, suggesting that the region rather than either cluster represents the phellem identity. The phellogen (cork cambium) and phelloderm identities were not clearly distinguishable in the dataset, indicating high similarity between these two periderm cell types. Cluster 4 was close to the periderm clusters, while it shares similar DEGs with cluster 20. Cluster 4- and 20-specific reporters not only showed expressions in the periderm, but also in vascular cells (Extended Data Fig. 1g). GO analysis indicated these clusters might consist of cells sensitive to temporal environmental change (Extended Data Fig. 1h).

New candidate promoters for overexpression analysis in Arabidopsis mature root

Next, we aimed to identify genes that are broadly and strongly expressed in the secondary tissue. We selected two genes with high number of reads in bulk RNA-seq data[4] and broad and strong expression in the scRNA-seq data of the mature root. *RIBOSOMAL PROTEIN S8A* (*RPS8A*) and *UBIQUITIN 11* (*UBQ11*) promoters drove *YFP* expression strongly and more ubiquitously in the secondary tissue than the commonly used promoter for overexpression, cauliflower mosaic virus 35S (*35S*)[4] (Extended Data Fig. 1i,j). Thus, these two promoters are useful for overexpression studies in root secondary tissues.

Xylem vessel maturation from committing to terminally differentiating cell and identification of fiber cells

Based on the expression of previously reported xylem-expressed genes (*NAC-DOMAIN PROTEIN 101* (*NAC101*)/*VASCULAR-RELATED NAC-DOMAIN 6* (*VND6*), *ATHB8*, *NAC-DOMAIN PROTEIN 043* (*NAC043*)/*NAC SECONDARY WALL THICKENING PROMOTING FACTOR1* (*NST1*))[5–7] (Extended Data Fig. 1b), the clusters above the meristematic cell band (5, 8, 11, 14, 16 and 17) were annotated as xylem clusters. In the dataset, the putative vessel cluster 17 was divided into two subclusters 17_1 and 17_2 (Fig. 1b). GO analysis of the differentially expressed genes (DEGs) revealed that cells in subcluster 17_1 were primarily involved in ribosome biosynthesis, while cells in subcluster 17_2 were undergoing intensive morphological modification, including secondary cell wall biogenesis (Extended Data Fig. 3a). These different GO terms support the idea that there are two stages of vessel differentiation in cluster 17. To further explore the identity of cluster 17, we generated transcriptional fluorescence reporter lines of genes enriched in cluster 17_1, *AT3G10080*, *LIKE AUXIN RESISTANT 2* (*LAX2*), *ACAULIS 5* (*ACL5*), *LACCASE 6* (*LAC6*), *PHY RAPIDLY REGULATED 2* (*PAR2*), *INDOLE-3-ACETIC ACID 6* (*IAA6*) and *PACLOBUTRAZOL RESISTANCE 5* (*PRE5*). Fluorescence signals in *AT3G10080*, *LAX2*, *ACL5*, and *LAC6* reporter lines were detected in expanding vessel cells and frequently in the cells on the xylem side of the cambium, which had not yet expanded (Extended Data Fig. 3b). In contrast, promoter activities of *PAR2*, *IAA6*, and *PRE5* were detected in the expanding vessels, but not in the xylem side of the cambium (Extended Data Fig. 3b). Because genes detected in the right half of subcluster 17_1 (such as *AT3G10080*, *LAX2*, and *ACL5*) tended to show the expression in the xylem-side cambium in their reporter lines, the small proportion of subcluster 17_1 would represent the earliest stage during commitment to vessel differentiation. Transcriptional reporter lines of genes enriched in subcluster 17_2 (*RHO OF PLANTS GUANINE NUCLEOTIDE EXCHANGE FACTOR* (*ROPGEF4*) and *AT4G27435*) showed strong expression in fully expanded vessels with bright cell wall staining (Extended Data Fig. 3c). In summary, cluster 17 is composed of vessels at different developmental stages; subcluster 17_1 represents vessel identity cells and expanding vessels, followed by differentiating vessels in subcluster 17_2.

Secondary xylem formation in *Arabidopsis* root and hypocotyl occurs in two phases. Initially, vascular cambium produces vessels and parenchyma cells, and when bolting is initiated, fiber cell and vessel formation begins[8]. Since the seedlings that we used for the scRNA-seq analysis had just initiated bolting, first fiber differentiation events were visible in their roots (Fig. 1a), and xylem fiber regulator *NAC043*/*NST1*[7] showed high expression in clusters 14 and 16 (Extended Data Fig. 1b). Additionally, the promoter activity of cluster 14- and 16-enriched gene, *CLAVATA3/EMBRYO SURROUNDING REGIONRELATED 46* (*CLE46)*, was highly detected in differentiating xylem fiber cells (Extended Data Fig. 4a), further supporting the idea that xylem fiber cells are enriched in clusters 14 and 16.

Secondary sieve element and companion cell maturation resemble the process in primary development

In the primary root, the phloem tissue consists of conductive sieve elements and companion cells[9], and *DOF2.4*/*PEAR1* is a key regulator and marker in conductive phloem specification[10]. Since *PEAR1* was reported to be expressed in the phloem-side of the cambium[6], we examined *PEAR1* in our dataset. *PEAR1* was detected in the lower part of the meristematic cell band, preferentially cluster 15, and cluster 21; its transcriptional reporter line showed the highest expression in the phloem-side cambium and the weaker signals in the phloem region along with the conductive phloem formation, suggesting that the *PEAR1*-expressing clusters include phloem identity cells and subsequent daughter cells (Extended Data Fig. 5d). To annotate the conductive phloem clusters in more detail, we investigated the genes that act as downstream of *PEAR1* in the primary phloem[11]. In primary phloem development, *PEAR1* promotes the bifurcation of the procambium and the sieve element lineage by activating *ROPGEF* and *ALTERED PHLOEM DEVELOPMENT* (*APL*) expression, respectively[11]. We found that *ROPGEF2* and *ROPGEF3* showed specific expressions in a subgroup of cells in cluster 15 (Extended Data Fig. 5a). The transcriptional reporter lines of these genes showed strong fluorescence signals in secondary phloem cells undergoing formative division to form sieve elements and

companion cells (Extended Data Fig. 5a). These results imply common factors regulating asymmetric cell divisions in primary and secondary phloem formation. *APL* and *SUCROSE-PROTON SYMPORTER 2* (*SUC2*), known as conductive-phloem marker genes[12,13], were highly detected in cluster 21 and 19 (Extended Data Fig. 1b). Reporter analysis of genes specific to cluster 21 (*SYTD*, *MAPKK KINASE 20* (*MAPKKK20*) and *AT5G48060*) or cluster 19 (*ETHYLENE-RESPONSIVE ELEMENT BINDING FACTOR 14* (*ERF14*), *AT2G48090*[14], and *ISOPENTENYLTRANSFERASE 3* (*IPT3*)[15]) confirmed that cluster 21 and 19 consist of sieve elements and companion cells, respectively (Extended Data Fig. 5b,c).

Lineage tracing of mature phloem parenchyma cells

To investigate the transition of mature phloem parenchyma cells into periderm cells, we performed lineage tracing analysis. We induced single-cell YFP clones in the procambium within a few days after the initiation of secondary growth and treated the seedlings with JA or SA for three weeks. Among 88 vascular sectors, four sectors showed one cell or one cell file invasion into the periderm upon SA treatment (one cell invasion; 3 out of 88 sectors, one cell file invasion; 1 out of 88 sectors), whereas no sector showed such transition in mock-treated roots (none out of 47 sectors) (Extended Data Fig. 7b). Upon JA treatment, five out of 142 sectors showed two cells or an entire cell file invasion into the periderm (two cell invasion; four out of 142 sectors, cell file invasion; one out of 142 sectors) (Extended Data Fig. 7c). Conversely, mock-treated roots for JA treatment did not show any sectors with more than two cell invasions (75 sectors). Only three of them showed sectors with one cell invasion. Abscisic acid (ABA), another stress hormone which typically inhibits growth, failed to accelerate the transition (none out of 54 sectors), despite being able to inhibit radial growth.

Our data showed that 8-day JA treatment promotes more frequent transition events (32 out of 314 sectors, 10.08% with JA; 11/269 sectors, 4.20% Mock) than 3-week treatment (Fig. 3g, Extended Data Fig. 7l). This rather surprising finding can be explained by the way we could confidently trace only the recent transition events. In both short- (8 day) and long-term JA (3 weeks) treatment, we considered only small sector invasions into the periderm for the analysis (such as Fig. 4e), since we could not be sure whether the large sector invasions were result of a single recombination event, or two separate recombination events in adjacent cells located in both side of the clonal boundary. Thus, especially in the 3 weeks tracing experiments we excluded clones in which transitions were initiated at the beginning of JA treatment. In conclusion, with our lineage tracing method, we can confidently trace only rather recent transition events. Since the frequency of the transition events after 8 days tracing is markedly higher than after 3 weeks, we can assume that the majority of the transition events caused by JA treatment occurs during the first days of the treatment. Transitions during 1 to 3 weeks of JA exposure appears to be rare according to our 3 weeks lineage tracing analysis. Thus, the frequency in 3-week lineage tracing analysis is an underestimation of transition events during the whole 3 weeks period. Altogether, our results indicated that JA treatment accelerates the transition of mature phloem into periderm.

References

1. Zhang, T.-Q., Chen, Y. & Wang, J.-W. A single-cell analysis of the Arabidopsis vegetative shoot apex. *Dev. Cell* 56, 1056-1074.e8 (2021).

2. Eswaran, G. *et al.* Identification of cambium stem cell factors and their positioning mechanism. *Science* 386, 646–653 (2024).

3. Xiao, W. *et al.* Pluripotent Pericycle Cells Trigger Different Growth Outputs by Integrating Developmental Cues into Distinct Regulatory Modules. *Curr. Biol.* 30, 4384-4398.e5 (2020).

4. Ye, L. *et al.* Cytokinins initiate secondary growth in the Arabidopsis root through a set of LBD genes. *Curr. Biol.* 31, 3365-3373.e7 (2021).

5. Kubo, M. *et al.* Transcription switches for protoxylem and metaxylem vessel formation. *Genes Dev.* 19, 1855–1860 (2005).

6. Smetana, O. *et al.* High levels of auxin signalling define the stem-cell organizer of the vascular cambium. *Nature* 565, 485–489 (2019).

7. Mitsuda, N. *et al.* NAC Transcription Factors, NST1 and NST3, Are Key Regulators of the Formation of Secondary Walls in Woody Tissues of Arabidopsis. *Plant Cell* 19, 270–280 (2007).

8. Ragni, L. *et al.* Mobile Gibberellin Directly Stimulates Arabidopsis Hypocotyl Xylem Expansion. *Plant Cell* 23, 1322–1336 (2011).

9. Shahan, R. *et al.* A single-cell *Arabidopsis* root atlas reveals developmental trajectories in wild-type and cell identity mutants. *Dev. Cell* 57, 543-560.e9 (2022).

10. Miyashima, S. *et al.* Mobile PEAR transcription factors integrate positional cues to prime cambial growth. *Nature* 565, 490–494 (2019).

11. Roszak, P. *et al.* Cell-by-cell dissection of phloem development links a maturation gradient to cell specialization. *Science* 374, eaba5531 (2021).

12. Bonke, M., Thitamadee, S., Mähönen, A. P., Hauser, M.-T. & Helariutta, Y. APL regulates vascular tissue identity in Arabidopsis. *Nature* 426, 181–186 (2003).

13. Truernit, E. & Sauer, N. The promoter of the Arabidopsis thaliana SUC2 sucrose-H+ symporter gene directs expression of β-glucuronidase to the phloem: Evidence for phloem loading and unloading by SUC2. *Planta* 196, 564–570 (1995).

14. Otero, S. *et al.* A root phloem pole cell atlas reveals common transcriptional states in protophloem-adjacent cells. *Nat. Plants* 8, 954–970 (2022).

15. Takei, K. *et al.* AtIPT3 is a Key Determinant of Nitrate-Dependent Cytokinin Biosynthesis in Arabidopsis. *Plant Cell Physiol.* 45, 1053–1062 (2004).

Supplementary Table 1: Necessary reported information to allow evaluation and repetition of a plant single-cell/nucleus experiment.

Supplementary Table 2: Top differentially expressed gene list (DEGs) for each cluster or cluster combination versus the rest of the cells.

Supplementary Table 3: Gene Ontology analysis of top differentially expressed gene in each cluster.

Supplementary Table 4: Reporter summary.
