# Supplementary material for: The dynamic and diverse nature of parenchyma cells in the Arabidopsis root during secondary growth
Source: Nat Plants. 2025 Mar 26;11(4):878–90. doi: 10.1038/s41477-025-01938-6 (PMC12014502; doi:10.1038/s41477-025-01938-6)
Supplement: Supplementary file 3 — Necessary reported information to allow evaluation and repetition of a plant single-cell/nucleus experiment. [file 41477_2025_1938_MOESM3_ESM.pdf]

Supplementary Table 1: Necessary reported information to allow evaluation and repetition of a plant single-cell/nucleus experiment.

	Details	Experimental information
		Secondary root growth
Biological material	Species	*Arabidopsis thaliana*
	Accession	Col-0
	Genotype	*pPXY:erYFP* (Col-0)
	Tissue type	Root secondary tissue
	Detailed growth conditions	Details described in materials & methods
	Harvest conditions	Room temperature
Sample preparation	Isolation protocol	Details described in materials & methods
	Tissue dissection	Details described in materials & methods
	Fixation	-
	Cell/nuclei enrichment	FACS (BD Aria II)
	Total sample preparation time	~3.5h (1h incubation in enzyme solution)
	Estimated cell/nuclei number loaded	16,000 cells
	Instrument/Method/Kit	Chromium Next GEM Single Cell 3'Kit v3
	Cell viability test	-

Libraries	Library construction	According to manufacturer's instructions. 11 cycles were used for cDNA amplification and 12 for index PCR
	Amplification method	-
	End bias	3'
Sequence results	Instrument/method	Illumina HiSeq 4000
	Library layout/paired-end	Single index
	N° sequenced reads	577,256,270
Raw data	Reference genome	TAIR10
	Annotation version	release 40
	Mapping method (incl. software, customized settings)	Cellranger 6.1.2
	Mapping efficiency	87.9%
	Sequencing saturation	46.1%
	Estimation of ambient RNA	-
	Imputation method and settings	-
Processed data	N° captured cells	17,140
	N° high quality cells	11,760
	Filter criteria: % mitochondrial reads/cell or nucleus	Mitochondrial % < 15%
	Filter criteria: % chloroplast reads/cell or nucleus	Chloroplast % < 15%

	Filter criteria: Minimum N° UMI/cell or nucleus	950 < genes < 8,500<br>4,000 < UMIs < 84,000
	N° total detected transcripts	25,048
	Doublet rate	-
	Replicate comparisons	-
	Batch correction method for merging (incl. reasoning for batch correction)	-
	Additional processing	-
**Validation**	Method of automatic annotation of clusters	-
	Method of manual annotation (markers, gene function info)	Marker lines, gene function information
	Verification in planta (e.g. Number of markers used for validation)	93 marker lines
Data availability	Analysis scripts & codes (GitHub)	
	Excel Tables DEG for each cluster	
	Objects/count matrix in repository (which one, where?)	NCBI GEO GSE270140
	On-line tool/browser URL	www.single-cell.be/plant
	Cell-level metadata table	
**Additional**	additional comments from the authors	
